# When the Past Fades: Detecting Phylogenetic Signal with SatuTe

**DOI:** 10.1093/molbev/msaf090

**Published:** 2025-05-27

**Authors:** Cassius Manuel, Enes Sakalli, Heiko A Schmidt, Carme Viñas, Arndt von Haeseler, Christiane Elgert

**Affiliations:** Center for Integrative Bioinformatics Vienna, Max Perutz Labs, University of Vienna, Medical University of Vienna, Dr. Bohr Gasse 9, Vienna A-1030, Austria; Center for Integrative Bioinformatics Vienna, Max Perutz Labs, University of Vienna, Medical University of Vienna, Dr. Bohr Gasse 9, Vienna A-1030, Austria; Vienna BioCenter PhD Program, Doctoral School of the University of Vienna and Medical University of Vienna, Vienna A-1030, Austria; Center for Integrative Bioinformatics Vienna, Max Perutz Labs, University of Vienna, Medical University of Vienna, Dr. Bohr Gasse 9, Vienna A-1030, Austria; Faculty of Mathematics and Statistics, Polytechnic University of Catalonia, Barcelona, Spain; Center for Integrative Bioinformatics Vienna, Max Perutz Labs, University of Vienna, Medical University of Vienna, Dr. Bohr Gasse 9, Vienna A-1030, Austria; Ludwig Boltzmann Institute for Network Medicine, University of Vienna, Augasse 2-6, Vienna A-1090, Austria; Center for Integrative Bioinformatics Vienna, Max Perutz Labs, University of Vienna, Medical University of Vienna, Dr. Bohr Gasse 9, Vienna A-1030, Austria

**Keywords:** branch support, phylogenetic inference, phylogenetic signal, saturation, phylogenetic information, tree of life

## Abstract

In phylogenetics, the phenomenon of saturation is well known, although its influence on tree reconstruction lacks a systematic and well-founded method. Here, we propose a new measure of the phylogenetic information shared between two subtrees connected by a branch in a phylogeny. This measure generalizes the concept of saturation between two sequences to a theory of saturation between subtrees, whose implementation we provide as the versatile program SatuTe. We describe different usages of SatuTe, identifying which branches in a tree are phylogenetically informative and which alignment regions support a given branch. As an example, we discuss the Tree of Life reconstruction from ribosomal proteins and the 16S rRNA gene, with emphasis on the two-domain versus three-domain hypotheses. For the branch leading to Eukaryota, we show that most ribosomal proteins contain a strong phylogenetic signal, whereas some regions of the 16S rRNA gene have lost phylogenetic information. Thus, SatuTe opens new insights into phylogenetic inference and complements standard phylogenetic analysis.

## Introduction

Model-based phylogenetic inference using a Maximum-Likelihood Estimate has become common practice, largely due to the availability of phylogenetic software like PHYLIP (Felsenstein 2005), PhyML (Guindon et al. 2010), RAxML (Stamatakis 2014), or IQ-Tree (Minh et al. 2020). This practice involves inferring a phylogenetic tree from a multiple sequence alignment and subsequently validating it using various statistical methods, such as the aLRT to evaluate the reliability of short branches (Anisimova and Gascuel 2006). A popular method, the phylogenetic bootstrap, provides a support value for each branch in the tree, where a low bootstrap support value indicates that the branch has a poor reproducibility (Felsenstein 1985). Conversely, branches with high bootstrap support values are considered as evidence of a real phylogenetic signal, justifying the separation between the two groups in the tree. Implicit to all these analyses is the assumption that the alignment contains sufficient phylogenetic information to conclude that the branch displays a historical relationship (Ho and Jermiin 2004).

However, there is little to no phylogenetic information in two cases. The first case occurs when sequences have accumulated only a few substitutions over time, resulting in short branch lengths in a phylogenetic tree. The second case arises when too many substitutions obscure true genetic distances (Philippe et al. 2011). This phenomenon, known as saturation, occurs when so many substitutions have accumulated that the alignment no longer provides reliable phylogenetic information. For two sequences, an alignment as a whole is considered saturated when it becomes impossible to determine whether the sequences are homologous or merely randomly related to each other (Brown et al. 1982; Philippe and Forterre 1999; Jermiin and Misof 2020). When two sequences are saturated, the evolutionary distance between them, measured in substitutions per site, is so extensive that their evolutionary relationship cannot be justified. Consequently, linking them with a branch to create the smallest possible tree lacks phylogenetic significance.

For alignments with more than two sequences and a tree with many leaves, the question arises whether branches are subject to saturation and, more importantly, how to measure saturation. If we detect saturation for a branch, then the phylogenetic signal for the two groups, separated by the branch, is so small that an evolutionary relationship among them is not justifiable.

Leaving aside the maximum-parsimony test (Archie 1989) and ad hoc analyses (Dávalos and Perkins 2008; Liu et al. 2014), Duchêne et al. (2021) proposed a saturation index that measures the entropy of alignment sites relative to the maximum entropy achieved when states are chosen at random (Xia et al. 2003). While effective for assessing saturation at the alignment level, this index is global, relying solely on the alignment data without considering the phylogenetic tree structure. Consequently, it cannot detect saturation at the level of individual branches within a phylogenetic tree.

Moreover, in practice, saturation may not affect the entire alignment uniformly; instead, specific regions, such as third codon positions, may show saturation while other parts remain well conserved (Salemi 2009). This suggests that the degree of saturation for a branch can vary based on the regions considered.

Alternative approaches to describe phylogenetic informativeness, acknowledging that phylogenetic information is not present when sequences are either too similar or saturated, were proposed (e.g. Yang 1998; Townsend 2007; Townsend et al. 2012; Klopfstein et al. 2017; Dornburg et al. 2019). Based on simulations, these methods determine the best evolutionary rate, for which the phylogenetic information is optimal. For example, Townsend (2007) compared the probabilities of characters supporting true versus incorrect trees in a four-taxon tree, considering variables such as the interior branch length *t* and the total rate of evolution *λ*. While insightful, it is limited on small-tree computations, which are difficult to generalize to larger trees. Additionally, the method identifies an optimal rate $\hat{\lambda }$ but does not establish a formal threshold to distinguish informativeness from saturation.

Here, we introduce SatuTe (*Satu*ration *Te*st), a new tool that fills a critical gap in the phylogenetic toolkit by detecting when sequence saturation hinders accurate inference of evolutionary relationships. For a phylogenetic tree and an alignment, SatuTe evaluates whether the alignment provides enough phylogenetic information to justify the branches in the tree. SatuTe embeds the analysis of saturation between two sequences into a general framework that handles multiple sequences and a tree. Thus, SatuTe opens a new avenue for studying the relationship between alignments and trees and helps to consolidate evolutionary conclusions.

In the following, we present the theoretical foundations of SatuTe. We explain the test designed to evaluate the null hypothesis of saturation against the alternative hypothesis indicating the presence of a phylogenetic signal. Alternatively, the *P*-value computed from our test statistic indicates the strength of evidence against the null hypothesis of saturation for a branch. We then evaluated the performance of SatuTe on simulated alignments and investigate its applicability for typical phylogenetic questions. These questions focus on deciding whether a branch in the tree is supported by the phylogenetic signal of the alignment. Lastly, we use SatuTe to examine real data, analyzing the phylogenetic signal of ribosomal protein and 16S rRNA alignments for the Tree of Life (ToL). As a case study, we analyze the branch connecting Eukaryota with the rest of the tree to shed new light on the ongoing debate between the two-domain hypothesis (Hug et al. 2016; Williams et al. 2020) and the three-domain hypothesis (Woese et al. 1990; Gribaldo et al. 2010).

## Results

### Foundations of SatuTe

To keep the explanation simple, we present the theory and statistical method using DNA, though they are equally applicable to amino acid sequences. For further details, we refer to Methods and supplementary material C, Supplementary Material online.

**Fig. 1.**
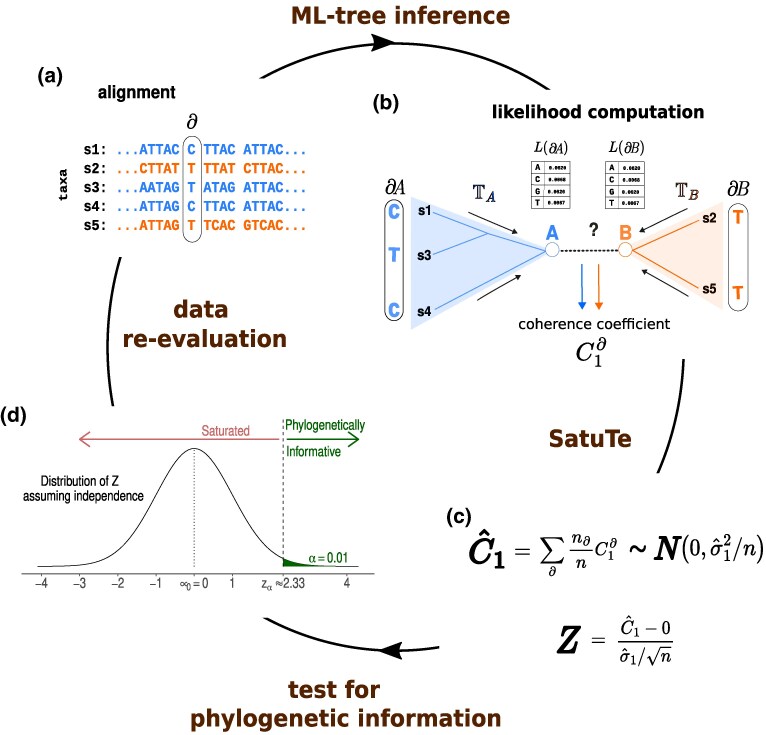
SatuTe in a nutshell. a) Five-taxon DNA alignment where each column represents a pattern ∂. b) The branch *AB* splits the five-taxon tree into subtree $T_{A}$ with sequences s1,s3,s4 and subtree $T_{B}$ with sequences s2,s5. Additionally, branch *AB* splits each pattern, such as ∂=CTTCT, into subpatterns ∂A=CTC and ∂B=TT. These subpatterns are used to compute the likelihood vectors L(∂A) and L(∂B), which determine the coherence coefficients. c) The average coherence ${\hat{C}}_{1}$ is approximately normally distributed, with expectation zero and standard error ${\hat{\sigma }}_{1}/\sqrt{n}$, if the subtrees $T_{A}$ and $T_{B}$ are independent. The z-transform of the average coherence is then used to test for phylogenetic information. d) The null hypothesis of independently evolving subtrees is rejected at the significance level *α* if $Z>z_{\alpha }$. Rejection implies phylogenetic signal. Thus, the subtrees share a common history and are connected by a branch.

Figure 1a displays an alignment of length *n*, where the columns of the alignment represent the *n* site patterns ∂, while each of the *m* rows represents a nucleotide sequence of the taxa comprising the leaves of a phylogenetic tree. We assume the GTR model, a reversible and stationary model of sequence evolution with rate matrix Q and stationary nucleotide distribution π (Tavaré 1986). The matrix $\text{Diag}(\pi )P(t)=\text{Diag}(\pi )e^{Q\cdot t}$ describes the probability of each nucleotide pair if *t* substitutions per site (branch length) have occurred.

For the model of sequence evolution and the phylogenetic tree, we ask if the branch *AB* (Fig. 1b) is supported by the phylogenetic signal provided by the alignment. To this end, we compute for site pattern ∂ the (partial) likelihood vectors L(∂A) and L(∂B) at nodes A and B (Felsenstein 1981), where ∂A and ∂B are the subpatterns of ∂ induced by branch *AB*. The probability of observing pattern ∂ of the alignment is computed as


(1) $$P(\partial |t)={L(\partial A)}^{T}\text{Diag}(\pi )P(t)L(\partial B),$$

where *t* is the branch length of *AB*. Moreover, the reversibility of the substitution model allows the spectral decomposition of matrix Diag(π)P(t) (Levin and Peres 2017), expressed as


(2) $$\text{Diag}(\pi )P(t)=\pi \pi^{T}+\sum_{i=1}^{3}h_{i}h_{i}^{T}e^{\lambda_{i}t},$$

where $\lambda_{0}=0>\lambda_{1}\geq \lambda_{2}\geq \lambda_{3}$ are the eigenvalues of the rate matrix and $h_{k}$ are the corresponding left eigenvectors (i=1,2,3).

Substituting Equation (2) into (1), we derive the central equation


(3) $$P(\partial |t)=P(\partial A)P(\partial B)+\sum_{i=1}^{3}\langle L(\partial A),h_{i}\rangle \langle L(\partial B),h_{i}\rangle \;e^{\lambda_{i}t},$$

where ⟨⋅,⋅⟩ denotes the scalar product, and P(∂A)=⟨L(∂A),π⟩, P(∂B)=⟨L(∂B),π⟩ are the probabilities for patterns ∂A,∂B given subtrees $T_{A},T_{B}$, respectively. Note that the product P(∂A)P(∂B) describes the probability of pattern ∂ if the subtrees are independent from each other.

Rewriting Equation (3) in a more compact form, and assuming all eigenvalues are different, we obtain


(4) $$\frac{P(\partial |t)}{P(\partial A)P(\partial B)}=1+\sum_{i=1}^{3}C_{i}^{\partial }e^{\lambda_{i}t},$$

where $C_{i}^{\partial }$ is given by


(5) $$C_{i}^{\partial }=\left\langle \frac{L(\partial A)}{P(\partial A)},h_{i}\right\rangle \left\langle \frac{L(\partial B)}{P(\partial B)},h_{i}\right\rangle .$$

Equation (4) shows that with increasing branch length *t*, the ratio of the probability for pattern P(∂∣t) for the tree and the product of the probabilities for the two subpatterns converges exponentially quickly to one, since the nonzero eigenvalues $\lambda_{i}$ are negative.

Conversely, if $T_{A}$ and $T_{B}$ are independent, then the sum in Equation (4) must equal zero for all branch lengths *t*. While we cannot expect that this be true for an individual pattern ∂, we show that the expectation of any coefficient $C_{i}^{\partial }$, when summed over all patterns, equals zero if $T_{A}$ and $T_{B}$ are independent. We call $C_{i}^{\partial }$ the coherence coefficient (for the nonzero eigenvalue $\lambda_{i}$). Because the second largest eigenvalue $\lambda_{1}$ dominates the sum in Equation (4), we will restrict our analysis to the coherence coefficient $C_{1}^{\partial }$.

The expected coherence coefficient is estimated from the alignment and the tree as the average ${\hat{C}}_{1}=(1/n)\sum_{s=1}^{n}C_{1}^{\partial_{s}}$ for the *n* site patterns $\partial_{s}$. According to the central limit theorem, the average coherence coefficient is approximately normally distributed (Fig. 1c) with expectation 0 and standard error $\sigma_{1}/\sqrt{n}$, which can be estimated from the data (see supplementary material Methods M.1, Supplementary Material online). ${\hat{C}}_{1}$ can be z-transformed to obtain a normalized z-score *Z*. The null hypothesis that no phylogenetic signal exists to justify branch *AB* is rejected if the z-score *Z* is larger than the critical value $z_{\alpha }$ of a specified significance level *α* (Fig. 1d).

### SatuTe Applied to Simulated Data

**Fig. 2.**
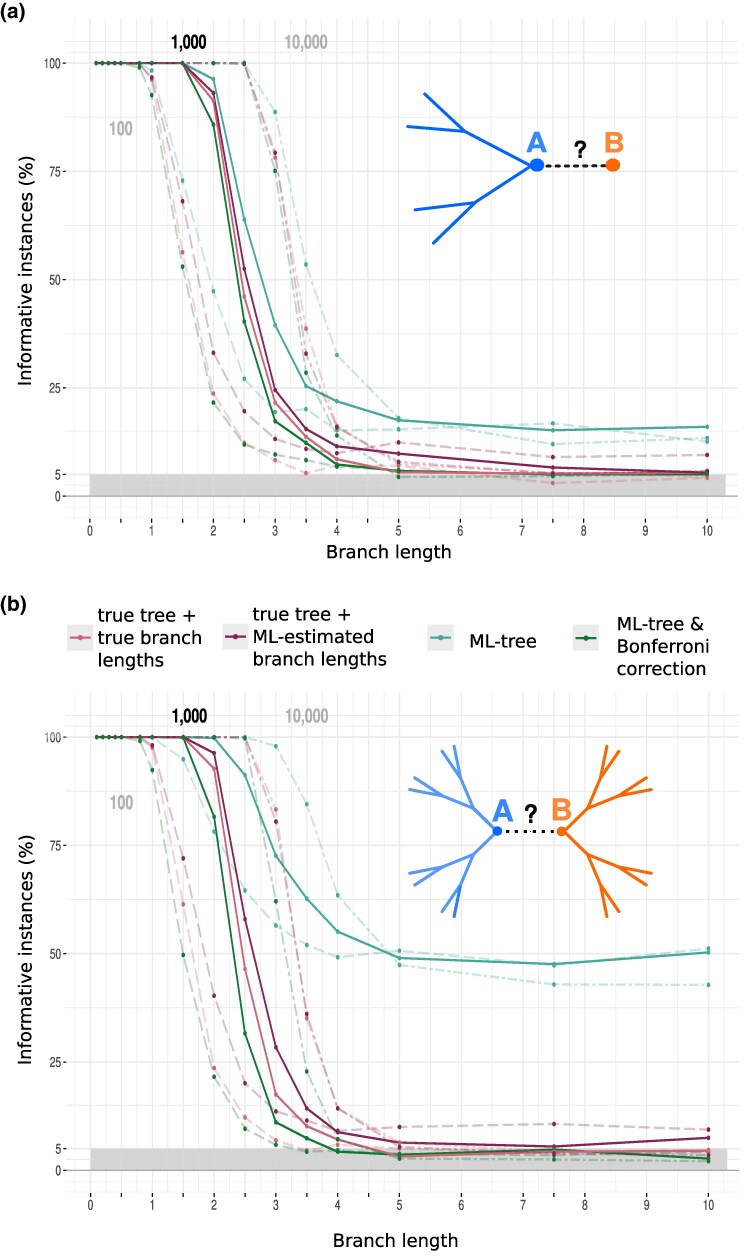
Performance of SatuTe in simulations. The plot shows the fraction of phylogenetically informative simulated alignments for branch *AB* and various analysis scenarios: the true tree (pink), the true tree topology with ML-estimated branch lengths (purple), and the inferred ML-tree including branch lengths (blue). The green curves represent the percentage of informative instances after applying the Bonferroni correction to the light green instances. In a), an external branch of a five-taxon tree and in b), the central branch of a balanced 16-taxon tree is analyzed. Dashed, solid, and alternating dashed lines represent the results for 100, 1,000, and 10,000 sites, respectively.

We analyzed the performance of SatuTe for a wide variety of parameter selections, including different numbers of taxa (5 and 16), different alignment lengths (100, 1,000, 10,000), various branch types (internal, external), and different branch lengths for the branch *AB* of interest (Fig. 2). For each parameter combination, we simulated 1,000 alignments along the specified tree assuming the Jukes–Cantor (JC) model (Jukes and Cantor 1969). For details of the simulation, see supplementary material Methods M.4, Supplementary Material online.

In the *first scenario*, representing the statistically correct situation (Fig. 2, pink), the alignment is simulated along the true (simulation) tree with its true branch lengths. Subsequently, the phylogenetic signal of the simulated alignment was tested on the true tree (including the true branch lengths) for the external branch (Fig. 2a) and the internal branch (Fig. 2b). Regardless of the specific parameters, the fraction of simulated alignments that are phylogenetically informative for branch AB decreases from 100% to the significance level of 5% as the branch length increases. Thus, long branch lengths in the true evolutionary process resulted in lower rejection rates of the null hypothesis of subtree independence, leading to more instances where the subtrees lack a phylogenetic signal for *AB*. According to standard statistical theory, longer alignments preserve phylogenetic signal for longer branches, so the overall behavior of the curves follows a typical power curve and serves as a basis for other scenarios that deviate from this gold-standard.

A realistic application similar to the first scenario is the following. After reconstructing a phylogenetic tree from a long (phylogenomic) alignment, one might want to identify which genes or regions are informative for the branches of the reconstructed tree. To achieve this, we analyze the reconstructed tree and test to which extent the genes or regions, which constitute only a fraction of the overall alignment, are phylogenetically informative.

Another application of SatuTe arised when we have a widely accepted tree and a new alignment for a gene family that has not yet been investigated. In this case, we would infer the branch lengths for the new alignment and check which branches in the accepted tree are supported by the new alignment. In other words, we determine where the new alignment is phylogenetically informative within the accepted tree topology.

This is what we consider in our *second scenario* (Fig. 2, purple). Here, the true tree topology is known, but the branch lengths are maximum-likelihood (ML) estimates based on the alignment. Figure 2 shows that the purple curves are extremely close to the gold-standard (first scenario). For short sequences (100 sites), the test turns out to be too liberal, with an empirical rejection level of approximately 10%, but we conclude that this is still tolerable.

The *third scenario* (Fig. 2, blue) involves reconstructing an ML-tree for an alignment and then determining where in the ML-tree the same alignment is phylogenetically informative. This scenario, where the data are used twice—namely to infer the tree and then to investigate the phylogenetic signal of branches—is known as circular analysis in statistics. Such double-dipping into the data undermines statistical inference and may lead to an inflation of the type I error (Button 2019). This is actually what we observe in Fig. 2, where for long branches 15% of the alignments are tested as phylogenetically informative for the external branch (Fig. 2a) and 50% of alignments for the internal branch (Fig. 2b), regardless of the sequence length. The high rejection rate is explained by the observation that for long branches AB most of the reconstructed trees disagree with the true tree. Most of the inferred ML-trees link subtrees $T_{A}$ and $T_{B}$ via their external branches, resulting in an incorrect tree (supplementary material Table S1, Supplementary Material online). Thus, if subtrees have $m_{A}$ and $m_{B}$ leaves, then the tree with the highest likelihood among $m_{A}\cdot m_{B}$ trees is selected, which happens to be phylogenetically informative.

The *fourth scenario* shows that the Bonferroni adjusted significance level $\alpha_{\mathrm{adjust}}=\alpha /(m_{A}m_{B})$ provides power curves (Fig. 2, green) that are close to the gold-standard. However, if the subtrees contain a large number of similar sequences, then $\alpha_{\mathrm{adjust}}$ may become too small, resulting in a too conservative test under this correction (supplementary material Fig. S1, Supplementary Material online). Then it may help to reduce the number of leaves and to carry out additional simulations tailored for the specific question and data.

Figure 2 shows the results generated under the assumption that no model misspecification occurs. Since the computation of the coherence coefficient obviously depends on the selected model of sequence evolution, we also conducted a simulation study under conditions of model misspecification (see supplementary material A.3, Supplementary Material online). For this analysis, we evolved sequences under a GTR-model (Tavaré 1986) and then inferred an ML-tree assuming either a JC or Kimura two parameter (K2P) (Kimura 1980) model. In both cases we observe a too liberal test (supplementary material Fig. S4, Supplementary Material online), with too many alignments considered phylogenetically informative for the branch *AB* compared with the gold-standard. However, when we replaced the JC or K2P model by the F81 model (Felsenstein 1981), which incorporates the base composition of the alignment, SatuTe performed well and the power curves approach the gold-standard (supplementary material Fig. S4, Supplementary Material online).

In conclusion, under a reversible and stationary model of sequence evolution, the most crucial aspect for SatuTe’s performance is the accurate estimation of base composition rather than substitution rate parameters. For instance, in cases where alignment sequences show substantial compositional heterogeneity, an incorrect estimate of the nucleotide distribution—which serves as proxy for the stationary distribution—violates one assumption of SatuTe and influences the test outcome. Model violations are known to impact all stages of a phylogenetic analysis (Jermiin et al. 2020) and will compromise SatuTe’s performance. In such situations, the user should consider specially designed simulations to discuss possible pitfalls of SatuTe.

### Saturation and Accuracy of Phylogenetic Inference

**Fig. 3.**
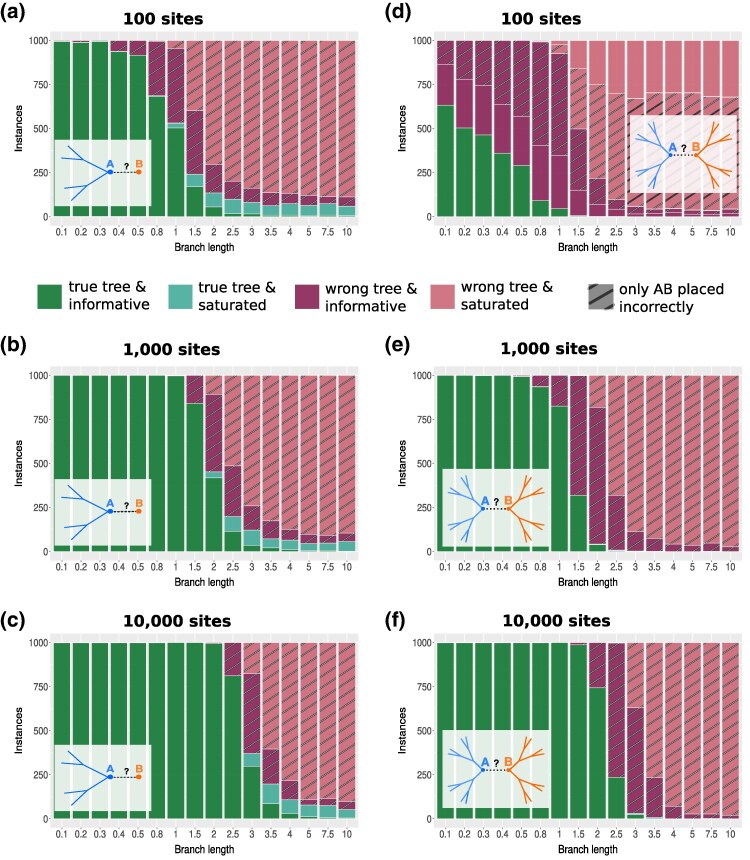
Saturation and accuracy. Further analysis of the ML-tree and Bonferroni correction scenario (dashed line, green) from Fig. 2. The left column presents results for the 5-taxon tree, while the right column shows results for the 16-taxon tree. Bar plots show the fraction of instances based on two conditions: correctly reconstructed trees (green shades) vs. incorrectly reconstructed trees (red shades) and whether branch *AB* is phylogenetically informative (dark shades) or saturated (light shades). Results are provided for datasets with 100 a, d), 1,000 b, e), and 10,000 c, f) sites, respectively. Instances of incorrect inference solely due to branch *AB* being misplaced between the subtrees $T_{A}$ and $T_{B}$—both correctly reconstructed as unrooted subtrees—are marked with hatched regions.

We explore whether SatuTe provides insights into the accuracy of phylogenetic inference. To this end, we further analyzed the data from the ML-tree and Bonferroni correction scenario presented in Fig. 2. Each instance was classified according to two criteria: correctly reconstructed trees (green shades) vs. incorrectly reconstructed trees (red shades) and whether branch *AB* was phylogenetically informative (dark shades) or saturated (light shades). The results are shown in Fig. 3, with the 5-taxon tree in the left column and the 16-taxon tree in the right column.

Intuitively, saturation is expected to reduce the accuracy of tree reconstruction. Figure 3 confirms this: As the length of branch *AB* increases, the proportion of correctly reconstructed trees (green) decreases. This trend is consistent across both trees and all sequence lengths. Moreover, as it is well known (see for example Huelsenbeck 1995; Pollock et al. 2002), accuracy improves with longer sequence lengths.

For correctly reconstructed ML-trees (light/dark green), the analysis of a five-taxon tree with a sequence length of 100 sites shows that for branch lengths shorter than 0.5 time units, the inferred tree is correct in at least 90% of the instances, and in all of these cases, branch *AB* is phylogenetically informative (dark green). However, with increasing branch length, the proportion of saturated branches (light green) increases. This shows that saturation alone does not necessarily imply an incorrect reconstruction, as correctly reconstructed trees can still contain saturated branches.

Conversely, if SatuTe indicates saturation, the inferred tree is likely to be incorrect (light red) for branch lengths longer than 3.5 in approximately 90% of instances for the 5-taxon tree and in all instances for the 16-taxon tree, specifically for sequence lengths of 100 or 1,000 sites.

In all incorrectly reconstructed trees (red shades), the branch *AB* is misplaced. Except for the 16-taxon tree with sequence lengths of 100 sites, the incorrect ML-tree results exclusively from the misplacement of branch *AB* between the subtrees $T_{A}$ and $T_{B}$, both of which are correctly reconstructed as unrooted subtrees (indicated by hatching in Fig. 3). Thus, if branch *AB* is identified as saturated (light red), removing *AB* would result in two correctly reconstructed subtrees. For a sequence length of 100 sites (Fig. 3d), removing the saturated branch would, at least partially, improve the accuracy of the reconstructed tree. Notably, whether branch *AB* is phylogenetically informative (dark red, dark green) cannot reliably determine the accuracy of the full-tree phylogenetic inference.

Furthermore, for trees with very short branches (supplementary material Fig. S3, Supplementary Material online) that are challenging to infer, the proportion of incorrectly reconstructed trees that cannot be attributed solely to the incorrect placement of branch *AB* increases (red, hatched regions). In these cases, the well-known influence of sequence length on accuracy plays a more substantial role than the saturation of branch *AB* itself. However, as shown in supplementary material Fig. S3, Supplementary Material online, with increasing sequence length, other reconstruction artifacts decrease, leaving the saturation of branch *AB* as the primary factor responsible for the incorrectly reconstructed trees.

The findings presented above provide a quantitative perspective on two long-standing challenges in phylogenetic research: selecting suitable outgroups for rooting an unrooted tree (Maddison et al. 1984; Sanderson and Shaffer 2002, and references therein) and identifying rogue taxa (Wilkinson 1996; Aberer et al. 2013).

Both the 5-taxon and 16-taxon tree scenarios highlight the difficulties of rooting an ingroup tree $T_{A}$ using $T_{B}$ as an outgroup. In the 5-taxon tree, a single taxon serves as the outgroup, while in the 16-taxon tree, the outgroup is composed of multiple taxa. Sanderson and Shaffer (2002) recommend using multiple outgroups to shorten the connecting branch and enhance the reliability of ingroup rooting. With SatuTe, we provide a method to evaluate the key parameter for reliable rooting: the phylogenetic information of the branch linking the ingroup to the outgroup. SatuTe estimates if the branch is phylogenetically informative and thus helps to select appropriate outgroups. Our findings show, when outgroups are too distantly related, reliably determining the root of the ingroup $T_{A}$ becomes impossible. In particular, if SatuTe indicates that the rooting branch between the ingroup and outgroup is saturated, the proposed rooting is likely to be incorrect.

Interestingly, the observed preference for external branches in the five-taxon tree, when the *AB* branch becomes long, does not align with the assumption (Sanderson and Shaffer 2002, p. 61) that distantly related outgroups ‘will often fall on the longest internal branch’, which in the five-taxon tree is always the internal branch.

Furthermore, the single taxon $T_{B}$ in the five-taxon tree also resembles a rogue taxon (Wilkinson 1996), whose unstable placement is driven by an elevated substitution rate (Sanderson and Shaffer 2002), as represented by the long *AB* branch. Thus, SatuTe may help identify such rogue taxa that are incorrectly placed due to saturated branches.

### SatuTe Investigates the ToL

**Fig. 4.**
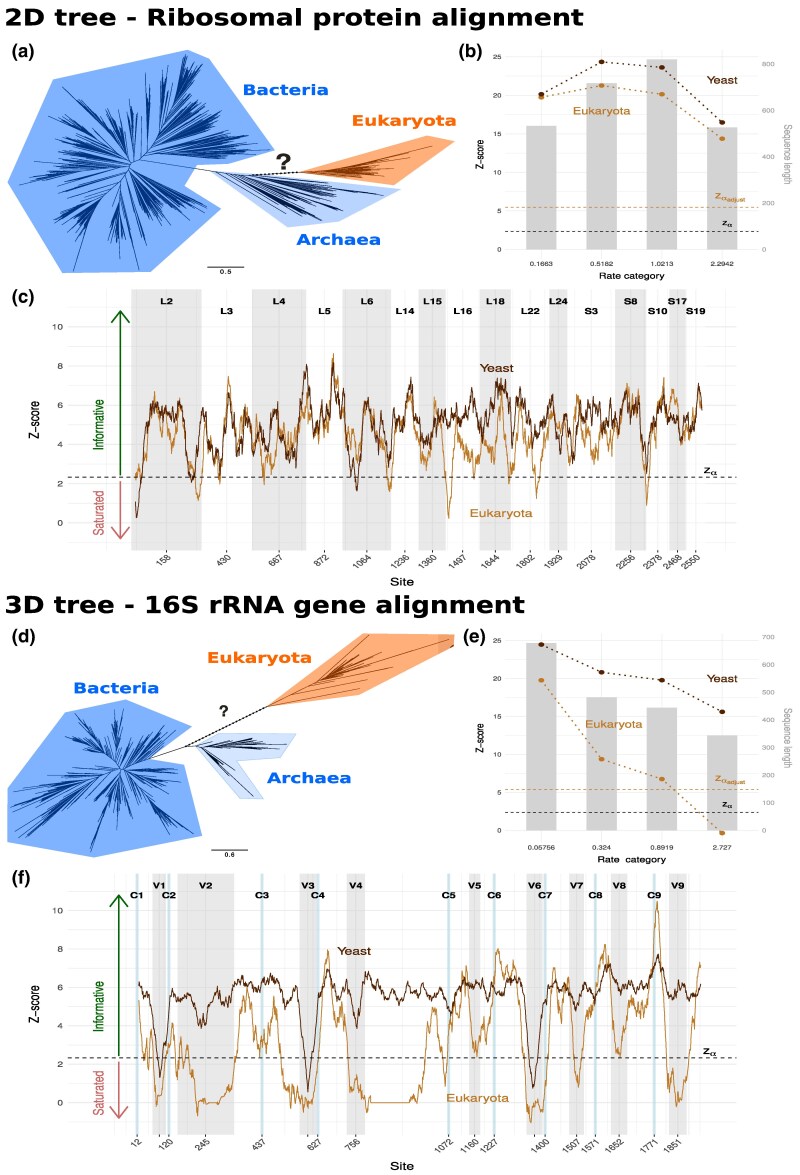
Per rate category and sliding-window analysis of selected branches: The branches leading to Eukaryota and yeast were analyzed. a) 2D tree with 3,083 taxa obtained from the ribosomal protein alignment and d) 3D tree with 1,871 taxa obtained from the 16S rRNA gene alignment. b, e) The per rate category z-scores are marked as dotted lines, and the estimated number of sites per category is represented by gray bars. For the Eukaryota branch, the Bonferroni adjusted critical value is shown. c, f) The progression of the z-score was calculated as a moving average for a sliding window with 36 sites.

We applied SatuTe to two alignments used to determine deep branching patterns in the ToL (Hug et al. 2016). The first alignment, comprising 16 ribosomal-subunit proteins, with a length of 2,596 amino acid sites and 3,083 taxa suggests the two-domain tree (2D ToL), in which the Eukaryota branch off within the Archaea (Fig. 4a). The second alignment, consisting exclusively of the 16S rRNA gene with 1,947 sites and 1,871 taxa, supports the three-domain tree (3D ToL), in which Archaea, Bacteria, and Eukaryota are each monophyletic (Fig. 4d). Depending on the alignments the 2D ToL or 3D ToL is inferred (see Hug et al. 2016).

To examine the phylogenetic information in both alignments, we inferred the branch lengths for both trees, using the LG+G4 model for the protein alignment and the GTR+G4 model for the 16S rRNAs (supplementary material Methods M.6, Supplementary Material online). We have focused our analysis on the models considered by the authors, as exploring alternative models is beyond the scope of our study.

Since the 2D ToL is based on an LG+G4 model and SatuTe is only applicable if all sites evolve at the same rate (supplementary material Methods M.3, Supplementary Material online), each alignment site was assigned to the rate category with highest posterior probability (Yang 1994). For sites within each rate category, we determined the phylogenetic signal for each branch according to the fourth scenario (ML-tree and Bonferroni correction). All 6,163 branches were phylogenetically informative for each of the four rate categories. Figure 4b displays the z-scores for the Eukaryota branch and for comparison an external branch leading to yeast.

For the 3D ToL, we inferred the branch lengths with the GTR+G4 model and repeated the above analysis. From the 3,739 branches, 107 branches were classified as saturated for sites from the highest rate category (rate 2.73). Among the saturated branches, there were 13 internal branches and one of them was the branch connecting Eukaryota with the rest of the tree (Fig. 4e). Thus, the fastest evolving sites in the 16S rRNA alignment do not provide support for the Eukaryota branch.

Given these results, we decided to focus on the branch that separates the Eukaryota from the rest in both the 2D and 3D ToL. Using SatuTe, we conduct a sliding-window analysis with a window size of 36 sites for both alignments (supplementary material Methods M.6, Supplementary Material online) to identify which regions of each alignment are phylogenetically informative for the Eukaryota branch. As the window length is small in relation to the total alignment, the analysis per window is similar to the first scenario of the simulations. Figure 4c and f shows the z-scores along the alignment. The ribosomal proteins show a clear phylogenetic signal for the entire alignment. The signal seems to be strong independently of the proteins. Only a few short regions exist where the phylogenetic signal has been lost (that is, z-score falls below the critical value $z_{\alpha }$). These regions are often found at the junction of two concatenated protein alignments. For the 3D ToL determined from the 16S rRNA alignment, we find many and sometimes very long alignment regions that are not phylogenetically informative (Fig. 4f). Saturated regions are mostly found in the so-called hypervariable regions V1 to V9 (Baker et al. 2004). On the other hand, from the conserved regions C1 to C9 in bacteria (Yang et al. 2016), regions C1 and C4 to C9 show phylogenetic signal, whereas C2 and C3 are almost saturated (for the Eukaryota branch). The conspicuously long phylogenetically uninformative regions 240 to 301 (z-score $<1\cdot 10^{-16}$) and 789 to 956 (z-score $<1\cdot 10^{-14}$) of the alignment have a low z-score because they are gappy and therefore provide little information about the substitution model.

The sliding-window analysis of the 16S rRNA revealed that 43.0% of the 1,912 windows show no phylogenetic signal for the Eukaryota branch. In the protein alignments, we only find 5.4% saturated windows. Even if the protein alignments have conserved significantly more phylogenetic information from early evolution, we currently cannot decide whether the 3D ToL is an artefact due to many saturated regions in the alignment. SatuTe does test for saturation but does not determine which tree is correct. In any case, some regions of the 16S rRNA provide little phylogenetic information for the placement of the Eukaryota branch in the 3D ToL, as is evident from the z-scores (Fig. 4f).

**Fig. 5.**
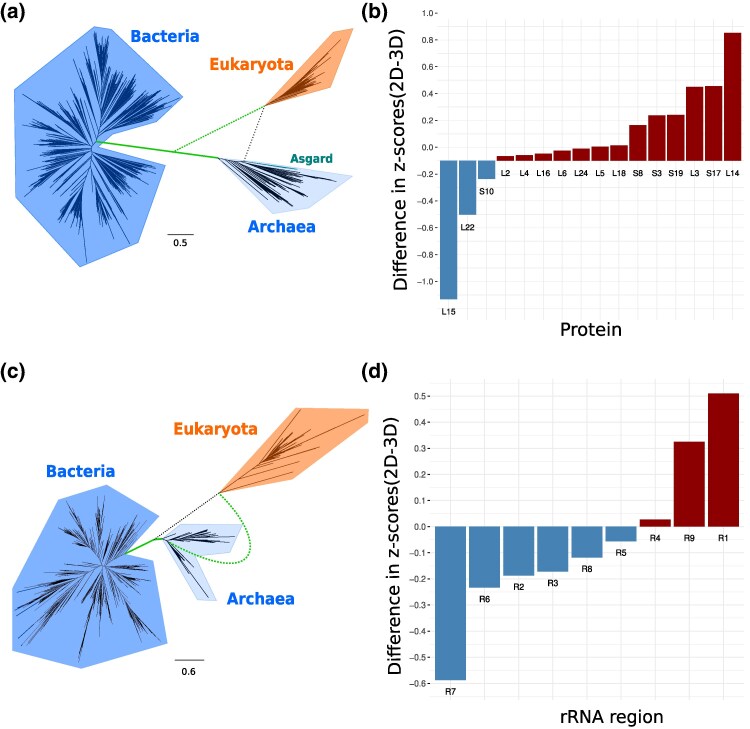
Differences between z-scores for the two placements of Eukaryota. The topology rearrangement of a) the protein-based 2D tree (Fig. 4a) into a 3D-tree and c) the 16S rRNA-based 3D tree (Fig. 4d) into a 2D-tree by replacing the black dashed branch with the green dashed branch. For each b) ribosomal protein and d) rRNA region, the z-score differences of the branch leading to Eukaryota between the 2D-tree and the 3D-tree are presented in ascending order.

Finally, we note that the z-score can tell us “how much” phylogenetic information is present for branches in different trees that induce the same partition of taxa. In this specific case, we calculated for the ribosomal proteins the z-scores for the Eukaryota branch in the 2D ToL and the 3D ToL (Fig. 5a). The tree in Fig. 5a shows the possible placements of the Eukaryota branch. To get the 3D ToL for the protein alignment, we pruned the Eukaryota subtree from the 2D-tree and regrafted it on the branch connecting Bacteria and Archaea. Then, following the second scenario, the ML-branch lengths were estimated for the rearranged tree and a LG+G4 substitution model. supplementary material Table S8, Supplementary Material online shows that the z-scores are significant for each protein, irrespective of 2D or 3D ToL. This suggests the presence of a phylogenetic signal that is independent of the trees. However, the 2D ToL z-scores are slightly larger except for proteins L15, L22, and S10 (Fig. 5b) and almost identical for L2.

We analyzed the 16S rRNA alignment in a similar way. Therefore, we performed the subtree and regrafting procedure on the 3D tree (Fig. 4d) to obtain the 2D ToL (Fig. 5c). The 16S rRNA alignment was divided into nine regions and for each region the z-score of the 2D ToL, 3D ToL, and the region-specific z-score differences were determined (supplementary material Table S9, Supplementary Material online). For six of the nine regions, the z-score difference is negative, i.e. these regions have a higher score in the 3D ToL (Fig. 5d).

Interestingly, the empirical distributions of the z-score differences for both alignments show no significant difference (Kolmogorov–Smirnov test: P-value=0.072). In summary, for the 16S rRNA alignment it is not clear which tree to prefer. However, the analysis yields valuable insights into the phylogenetic information that individual alignments (different protein or different regions) provide for branches in a phylogenetic tree.

## Discussion

SatuTe is a new method to quickly and efficiently compute the phylogenetic signal for every branch in a phylogenetic tree and a multiple sequence alignment. The practical applications of SatuTe are numerous and we have shown only some typical scenarios with simulated alignments and other applications based on alignments used in the discussion about the structure of the ToL (Cunha et al. 2017; Williams et al. 2020). The simulations show that SatuTe has all the properties of a good test and is also relatively robust when the ideal statistical conditions are not met. Our experiments (simulations, ToL) reflect typical applications of SatuTe. Many other analyses are conceivable. To this end, we developed the SatuTe program package, which can be seamlessly integrated into a phylogenetic pipeline with IQ-Tree2, as a good and flexible starting point for more in-depth studies. SatuTe generates a comprehensive output for each alignment site, enabling further in-depth analysis, such as user-specific investigations of subregions, structural elements, or other parts of an alignment. Additionally, the z-scores enable the comparison between branches in a tree or even between trees. We have presented the principle of this analysis in the ToL example. A more detailed analysis, which obviously depends on the data and the specific scientific questions, is beyond the scope of the paper. However, SatuTe offers the bioinformatic tools and the essential statistics to establish the groundwork for such an in-depth analysis.

That is why we did not resolve the discussion about the true phylogenetic relationship of Archaea, Bacteria, and Eukaryota. Our analysis clearly highlighted strengths and weaknesses of the alignments that led to the conflicting hypotheses about the ToL. SatuTe helps to come to a more objective decision which alignments to include or exclude in a phylogenetic study.

SatuTe may be seen as a complementary tool to bootstrap methods (Felsenstein 1985; Stamatakis et al. 2008; Hoang et al. 2017; Lemoine et al. 2018), which measure how strongly an alignment supports a split between two groups. In the classic bootstrap interpretation, a high bootstrap value is taken as a consistency value for the branch in the tree. SatuTe helps to determine whether the two groups are related, i.e. that a branch indicates a common evolutionary history or if the high bootstrap value simply reflects the dissimilarity between the groups (supplementary material Table S6, Supplementary Material online).

Furthermore, SatuTe provides insights into the quality of phylogenetic relationships for a given branch but cannot determine whether the overall tree is correct. When branches are so long that there is minimal phylogenetic information linking the subtrees, the relationship becomes unreliable. This often results in connections in the ML-tree that are nearly random among the many possible ways to link the two subtrees (see supplementary material Tables S2, S3 and S4, Supplementary Material online). Although SatuTe cannot determine whether a tree is reconstructed correctly, it can still provide useful insights by identifying potentially misrooted subtrees or distantly related rogue taxa when their connecting branches are found to be saturated.

The basic theory behind SatuTe gives additional information about the fading of the phylogenetic signal as the number of substitutions grows. As is well known (Mossel and Peres 2003; Levin and Peres 2017), the second largest eigenvalue describes the speed of the substitution model towards the stationary distribution. For the ToL discussion, the second largest eigenvalue of the LG+G4 model equals −0.27 and −0.83 for the GTR+G4 model. Therefore, the accelerated loss of phylogenetic signal along a branch under the GTR model, occurring three times faster offers at least a partial explanation for the preference of proteins in inferring deep phylogenies. This may also lead to a more systematic understanding for the usefulness of substitution models for inferring deep phylogenies (see Kapli et al. 2023).

Moreover, the computation of the coherence coefficient can be formally generalized for nonstationary and nonreversible substitution models using the π-scalar product (Levin and Peres 2017) between the likelihood vectors (Manuel 2022). However, dropping those assumptions leads to root identifiability issues. This implies that the test for saturation does not depend only on the parent node of the two subtrees, but also on the placement of the root. This complication leads to a slightly more technical test, that will be implemented in future versions of SatuTe.

In summary, SatuTe enables completely new possibilities for phylogenetic analysis. We were only able to demonstrate some typical applications that look promising. Our software package, SatuTe, allows even the uninitiated to quickly carry out the presented applications. Additionally, SatuTe provides a detailed output for each alignment position, which can then be analyzed for further questions.

### Estimates for SatuTe

The formal proofs of this section are to be found in supplementary material C, Supplementary Material online. Here we consider first the JC model, which has only two eigenvalues, with the second largest—of particular interest—having a multiplicity of three, i.e. $\lambda_{1}=\lambda_{2}=\lambda_{3}$. This case is analogous to the general case where the second largest eigenvalue has any multiplicity greater than one. For the JC model, the central Equation (4) can be written as


(6) $$\frac{P(\partial |t)}{P(\partial A)P(\partial B)}=1+\left(\sum_{k=1}^{3}\left\langle \frac{L(\partial A)}{P(\partial A)},h_{k}\right\rangle \left\langle \frac{L(\partial B)}{P(\partial B)},h_{k}\right\rangle \right)e^{\lambda_{1}t}.$$

Then the coherence coefficient corresponding to the second largest eigenvalue equals


$$C_{1}^{\partial }=\sum_{k=1}^{3}\left\langle \frac{L(\partial A)}{P(\partial A)},h_{k}\right\rangle \left\langle \frac{L(\partial B)}{P(\partial B)},h_{k}\right\rangle =\sum_{k=1}^{3}C_{1,k}^{\partial },$$

where $h_{k}$ are the left eigenvectors and $C_{1,k}^{\partial }$ are the coherence coefficients corresponding to the eigenvectors.

Given an alignment with *n* sites, we compute the empirical mean as an estimator for the random variable $C_{1}^{\partial }$,


(7) $${\hat{C}}_{1}\;:\;=\frac{1}{n}\sum_{s=1}^{n}C_{1}^{\partial_{s}}=\frac{1}{n}\sum_{s=1}^{n}C_{1,1}^{\partial_{s}}+C_{1,2}^{\partial_{s}}+C_{1,3}^{\partial_{s}}.$$

From the theory (supplementary material C, Supplementary Material online), we know that the expected value for the coherence coefficients $C_{1,k}^{\partial }$ is zero, under the null hypothesis of subtree independence. Due to the linearity of the expectation, this also holds for $C_{1}^{\partial }$, i.e. $E[C_{1}^{\partial }]=0$.

It is well known that for a sum of random variables, the variance is equal to the sum of all pairwise covariances:


$$\mathrm{Var}[C_{1}^{\partial }]=\mathrm{Var}\left[\sum_{k=1}^{3}C_{1,k}^{\partial }\right]=\sum_{i,j=1}^{3}\mathrm{Cov}\left(C_{1,i}^{\partial },\;C_{1,j}^{\partial }\right).$$

Note that $\mathrm{Cov}(C_{1,i}^{\partial },\;C_{1,i}^{\partial })=\mathrm{Var}[C_{1,i}^{\partial }]$. Furthermore, the covariance of two random variables can be calculated as the expected value of their product minus the product of their expected values, i.e. for our case:


$$\mathrm{Cov}\left(C_{1,i}^{\partial },\;C_{1,j}^{\partial }\right)=E\left[C_{1,i}^{\partial }\cdot C_{1,j}^{\partial }\right]-\underset{=0}{\underbrace{E[C_{1,i}^{\partial }]}}\cdot \underset{=0}{\underbrace{E[C_{1,j}^{\partial }]}}.$$

Since we already know that the expected values of $C_{1,k}^{\partial }$ is zero, the last term in the above equation is zero. To compute the expectation of the product of two coherence coefficients, we note under the null hypothesis of subtree independence that:


$$\begin{array}{rl} & E[C_{1,i}^{\partial }\cdot C_{1,j}^{\partial }]\\ & =E\left[\underset{=C_{1,i}^{\partial }}{\underbrace{\left\langle \frac{L(\partial A)}{P(\partial A)},h_{i}\right\rangle \left\langle \frac{L(\partial B)}{P(\partial B)},h_{i}\right\rangle }}\cdot \underset{=C_{1,j}^{\partial }}{\underbrace{\left\langle \frac{L(\partial A)}{P(\partial A)},h_{j}\right\rangle \left\langle \frac{L(\partial B)}{P(\partial B)},h_{j}\right\rangle }}\right]\\ & =E\left[\left\langle \frac{L(\partial A)}{P(\partial A)},h_{i}\right\rangle \left\langle \frac{L(\partial A)}{P(\partial A)},h_{j}\right\rangle \cdot \left\langle \frac{L(\partial B)}{P(\partial B)},h_{i}\right\rangle \left\langle \frac{L(\partial B)}{P(\partial B)},h_{j}\right\rangle \right]\\ & =\underset{=\sigma_{A,i,j}^{2}}{\underbrace{E\left[\left\langle \frac{L(\partial A)}{P(\partial A)},h_{i}\right\rangle \left\langle \frac{L(\partial A)}{P(\partial A)},h_{j}\right\rangle \right]}}\cdot \underset{=\sigma_{B,i,j}^{2}}{\underbrace{E\left[\left\langle \frac{L(\partial B)}{P(\partial B)},h_{i}\right\rangle \left\langle \frac{L(\partial B)}{P(\partial B)},h_{j}\right\rangle \right]}}, \end{array}$$

because the expected value is multiplicative under independence. Note that each expectation $\sigma_{A,i,j}^{2}$ and $\sigma_{B,i,j}^{2}$ is associated with one of the subtrees. If a subtree, e.g. $T_{A}$, consists of a single leaf, then $\sigma_{A,i,j}^{2}=\delta_{ij}$, where $\delta_{ij}$ is the Kronecker delta, defined as $\delta_{ij}=1$ if i=j and 0 if i≠j. Otherwise, the expectation $\sigma_{A,i,j}^{2}$ is estimated from the alignment using the empirical mean.

Therefore, the variance of the coherence coefficient $\mathrm{Var}[C_{1}^{\partial }]$ can be estimated from the alignment as


(8) $${\hat{\sigma }}_{1}^{2}=\sum_{i,j=1}^{3}{\hat{\sigma }}_{A,i,j}^{2}\cdot {\hat{\sigma }}_{B,i,j}^{2},$$

where


$${\hat{\sigma }}_{A,i,j}^{2}\;:\;=\left\{ \begin{array}{cc} \frac{1}{n}\sum_{s=1}^{n}\;\left\langle \frac{L(\partial_{s}A)}{P(\partial_{s}A)},h_{i}\right\rangle \left\langle \frac{L(\partial_{s}A)}{P(\partial_{s}A)},h_{j}\right\rangle ,\; & \;\text{for internal node A},\\ \delta_{ij}, & \;\text{for leaf A}, \end{array}\right.$$

and ${\hat{\sigma }}_{B,i,j}^{2}$ is constructed analogously.

In contrast to the JC model, the second largest eigenvalue of a typical GTR model has multiplicity one, which leads to a variance of the coherence coefficient $C_{1}^{\partial }$ of


$$\mathrm{Var}[C_{1}^{\partial }]=E[(C_{1}^{\partial }{)}^{2}]-0^{2}=E\left[{\left\langle \frac{L(\partial A)}{P(\partial A)},h_{1}\right\rangle }^{2}\right]\cdot E\left[{\left\langle \frac{L(\partial B)}{P(\partial B)},h_{1}\right\rangle }^{2}\right]$$

and a simpler estimator of the form


(9) $${\hat{\sigma }}_{1}^{2}={\hat{\sigma }}_{A,1}^{2}\cdot {\hat{\sigma }}_{B,1}^{2}$$

$${\hat{\sigma }}_{A,1}^{2}\;:\;=\left\{ \begin{array}{cc} \frac{1}{n}\sum_{s=1}^{n}\;{\left\langle \frac{L(\partial_{s}A)}{P(\partial_{s}A)},h_{1}\right\rangle }^{2},\; & \;\text{for internal node A},\\ \delta_{ij}, & \;\text{for leaf A}, \end{array}\right.$$

and ${\hat{\sigma }}_{B,1}^{2}$ is constructed analogously.

Note that we have presented the estimators for the coherence coefficients with respect to the second largest eigenvalue $\lambda_{1}$. However, this can also be done for any other eigenvalue in the same way.

### The Test for Phylogenetic Information

For a large alignment and independently evolving sites, our estimates are sufficiently accurate. According to the central limit theorem, ${\hat{C}}_{1}$ is normally distributed with an expectation of 0 and a variance of ${\hat{\sigma }}_{1}^{2}/n$. As in a typical one-sided z-test, we reject the null hypothesis of independently evolving subalignments at a given significance level *α* if the z-score *Z* satisfies


(10) $$Z\;:\;=\frac{{\hat{C}}_{1}-0}{{\hat{\sigma }}_{1}/\sqrt{n}}>z_{\alpha },$$

where $z_{\alpha }$ is the *α*-quantile of the standard normal distribution, satisfying $\text{Pr}(Z>z_{\alpha })=\alpha$.

If we reject independence, we conclude that the branch is phylogenetically informative, indicating that the subalignments share enough phylogenetic information to justify the connection in the tree. Otherwise, the branch is considered saturated.

The preceding theoretical results were derived under the assumption that the alignment and the placement of branch *AB* are independent of each other. However, in practice, the placement is usually inferred from the alignment itself. To roughly correct for the double-dipping, we can apply the multiple-test Bonferroni correction to the number of pairs of leaves used to place branch *AB*. Specifically, we adjust the significance level *α* to


(11) $$\alpha_{\mathrm{adjust}}=\frac{\alpha }{m_{A}\cdot m_{B}},$$

where $m_{A}$ and $m_{B}$ are the number of leaves in subtree $T_{A}$ and $T_{B}$, respectively.

### SatuTe with Rate Heterogeneity Model

If an evolutionary model with rate heterogeneity is used, each site is assigned to the rate category with highest posterior probability (Yang 1994). Then, for each category *c*, SatuTe employs the test for phylogenetic information as described in supplementary material Methods M.2, Supplementary Material online on the rescaled phylogenetic tree and the subalignment of the considered category. In this process, SatuTe determines for each category *c* the variance estimator ${\hat{\sigma }}_{1,c}^{2}$ according to Equation (8).

However, to test regions of an alignment, such as in a sliding-window analysis, we need to calculate an global variance estimator because the regions consist of sites belonging to different categories. For simplicity, consider four rate categories. Each category *c* has $n_{c}$ sites assigned. If the alignment has *n* sites, the global variance can be calculated as weighted average of the category variance estimates:


(12) $${\hat{\sigma }}_{1}^{2}\;:\;=\sum_{i=1}^{4}\frac{n_{c_{i}}}{n}\cdot {\hat{\sigma }}_{1,c_{i}}^{2}.$$

For an alignment window of size *w*, we perform the test according to M.2, using the average of all coherence coefficients over the *w* sites within the window for the estimate ${\hat{C}}_{1}$ and the standard error ${\hat{\sigma }}_{1}/\sqrt{w}$, where ${\hat{\sigma }}_{1}^{2}$ is obtained from Equation (12). We recommend a minimal sliding-window size of 30 sites, to ensure that the Central Limit Theorem is applicable.

### SatuTe in Simulations

We consider different simulation trees (see Table 1) with a branch *AB* connecting the subtrees $T_{A}$ and $T_{B}$. The branch length of *AB* was varied among {0.1,0.2,0.3,0.4,  0.5,0.8,1.0,1.5,2.0,2.5,3.0,3.5,4.0,5.0,7.5,10.0}. For each parameter combination, we simulated 1,000 DNA alignments with sequence length n=100,1,000,10,000 sites using Seq-Gen (v1.3.4; Rambaut and Grassly 1997) under the JC model. Each alignment was evaluated using IQ-Tree2 (Minh et al. 2020) and SatuTe with a significance level of α=0.05.

**Table 1.** Parameters used to analyze SatuTe in simulations.

	Fig. 2a	Fig. 2b
Simulation		
Tree:	5-taxon tree	16-taxon tree
Branch AB:	external, see Fig. 2a	internal, see Fig. 2b
Branches in $T_{A}$ & $T_{B}$:	all 0.2	drawn from EvoNAPS
With length range:	NA	0.04 to 0.2
Substitution model:	JC model	JC model
Evaluation		
Substitution model:	JC model	JC model
Bonferroni correction:	$\alpha_{\mathrm{adjust}}=0.05/4$	$\alpha_{\mathrm{adjust}}=0.05/64$

The fraction of phylogenetically informative alignments (rejecting the independence of subtrees $T_{A}$ and $T_{B}$) is plotted as a function of the branch length *AB* in the simulation tree for four different analyses:

1. Applying the test for phylogenetic information on the true simulation tree with fixed true branch lengths (pink), assuming the JC model for each alignment. (First scenario).
2. Applying the test for phylogenetic information on the true tree topology with ML-estimated branch lengths (purple), assuming the JC model for each alignment. (Second scenario).
3. Applying the test for phylogenetic information on the ML-inferred tree with estimated branch lengths (blue), assuming the JC model for each alignment. (Third scenario).
4. Using the Bonferroni correction $\alpha_{\mathrm{adjust}}$ (see Equation (11)) to correct the test results of the third scenario. (green).

The following table summarizes the most important aspects and differences of the simulation study for Fig. 2.

Branch lengths of the subtrees $T_{A}$ and $T_{B}$ in the 16-taxon trees were drawn between 0.04 and 0.2 or between 1/sequence−length and 0.2 from the distributions of internal and external branch lengths, respectively (see supplementary material Fig. S2, Supplementary Material online), as stored in the EvoNAPS database (Reden 2023, http://evonaps.cibiv.univie.ac.at).

Furthermore, each datapoint in Fig. 2b has been computed from an independent set of 1,000 alignments. In the rare cases where the inferred ML-tree did not recover the split induced by branch *AB* (i.e. the taxa of $T_{A}$ did not split from the taxa of $T_{B}$), the ML-tree was discarded. This determination of splits was done using TreeShredder (Bader 2023). Such removals occurred in only 13 of the 2,000 simulated alignments with n=100 and an *AB* branch length 0.1, specifically in 7 blue and 6 green instances.

### Saturation and Accuracy of Phylogenetic Inference

#### Protocol for Fig. 3

To understand if saturation influences the accuracy of phylogenetic inference, we further analyzed the data from the ML-tree and Bonferroni correction scenario presented in Fig. 2 (see supplementary material Methods M.4, Supplementary Material online). The instances in which branch *AB* was identified as informative were determined following the procedure outlined in supplementary material Methods M.4, Supplementary Material online.

The accuracy of the reconstructed ML-trees and the subtrees $T_{A}$, $T_{B}$ considered as unrooted subtrees was assessed using TreeShredder (Bader 2023). TreeShredder extracts the relevant splits from the reconstructed trees and the partial splits from the subtrees. A tree was correctly inferred if the set of all splits matched exactly those of the simulation tree; otherwise, it was classified as incorrectly inferred.

For incorrectly inferred trees, we assessed whether the incorrect inference was solely due to the misplacement of branch *AB* between the subtrees $T_{A}$ and $T_{B}$. This was verified by ensuring that all partial splits of subtrees $T_{A}$ and $T_{B}$ were correctly reconstructed as unrooted trees. In such cases, removing branch *AB* would result in two correctly reconstructed subtrees. In cases where the tree was reconstructed incorrectly for reasons beyond the misplacement of branch *AB*, errors were also present in the partial splits of the subtrees $T_{A}$ and/or $T_{B}$. In these situations, the sets of partial splits are incongruent with the structure of the subtrees $T_{A}$ and/or $T_{B}$.

Figure 3 shows the number of instances for all possible combinations—correctly vs. incorrectly reconstructed tree topology, whether branch *AB* was identified as informative or saturated, and whether the incorrect inference was solely caused by the misplacement of branch *AB*—was calculated and visualized.

### SatuTe and ToL

#### Protocol for Fig. 4

Hug et al. (2016) employed RAxML for tree reconstruction, using the LG+G4 model for the protein-based 2D tree (Fig. 4a) and the GTRCAT+G4 model for the rRNA-based 3D tree (Fig. 4d). For both alignments and corresponding trees, we used IQ-Tree2 to infer the branch lengths, using the LG+G4 model for the protein alignment and the GTR+G4 model for the 16S rRNA gene alignment. Note that the second largest eigenvalues of both substitution models have multiplicity 1. In both trees, we focused on the internal branch leading to Eukaryota and the external branch leading to yeast (*S. cerevisiae*).

Figure 4b, e: First, each site is assigned to the rate category with the highest posterior probability. The four subalignments resulting from the four categories where then tested for saturation. On average, sites associated with one category represent 25% of the alignment and may influence the tree reconstruction significantly. Thus, we used a conservative Bonferroni-corrected threshold $z_{\alpha_{\mathrm{adjust}}}$.

Figure 4c, f: Using the rate category assignment, SatuTe computed the coherence coefficient $C_{1}^{\partial }$ of each site and the variance ${\hat{\sigma }}_{1}^{2}$ as described in supplementary material Section M.3, Supplementary Material online. For each window, we perform the test according to supplementary material Section M.2, Supplementary Material online, using the average of all coherence coefficients over the 36 sites within the window. Since each 36-site window accounts for <2% of the total alignment, we did not consider the Bonferroni-correction necessary.

Figure 4c: For the annotation of the 16S rRNA gene alignment, we used the bacterial hypervariable regions annotated by Baker et al. (2004) and the small bacterial conserved regions annotated by Yang et al. (2016). Both annotations were initially based on *E. coli* reference sequences. We mapped these annotations to our alignment by accounting for the gaped sites present our dataset.

#### Protocol for Fig. 5

To investigate the impact of different tree topologies the phylogenetic information of protein sequence alignment, we focus on the branch leading to Eukaryota within both the 2D (Fig. 4a) and the rearranged 3D tree (Fig. 5a). Using SatuTe, we analyze these branches with the ribosomal protein alignment and each respective tree. The evolutionary model applied throughout is LG+G4 model. SatuTe outputs the calculation of z-scores for various proteins across both tree configurations and all four rate categories, with each protein region defined by an annotation file. These calculations follow the protocol detailed for Fig. 4c, and results are summarized in supplementary material Table S8, Supplementary Material online. Explicitly, ${\hat{C}}_{1}$ is computed using the $n_{P}$ sites of the tested protein, while ${\hat{\sigma }}_{1}^{2}$ is the global estimated variance described in Equation (12). We calculated the differences in the z-scores.

The 16S rRNA gene alignment was divided into nine distinct rRNA regions, as detailed in supplementary material Table S9, Supplementary Material online. For each rRNA region, we performed a similar analysis as for the protein alignment, focusing on the branch leading to Eukaryota within both the 3D (Fig. 4d) and the rearranged 2D tree (Fig. 5c).

## Supplementary Material

msaf090_Supplementary_Data

## Data Availability

The implementation of SatuTe is available at GitHub: https://github.com/Elli-ellgard/SatuTe. Additionally, all auxiliary scripts used for the various analyses are available in a separate GitHub repository, https://github.com/Elli-ellgard/SatuTe-example-analyses. Please note that the alignments were published previously and can be accessed through the original publications.
