# Supplementary material for: When the Past Fades: Detecting Phylogenetic Signal with SatuTe
Source: Mol Biol Evol. 2025 May 27;42(5):msaf090. doi: 10.1093/molbev/msaf090 (PMC12108095; doi:10.1093/molbev/msaf090)
Supplement: msaf090_Supplementary_Data [file msaf090_supplementary_data.zip › Supplements_SatuTe.pdf]

# Supplementary Information

**A SatuTe applied to simulated data** **34**
A.1 Placement of branch $AB$ in the ML-tree . . . . . . . . . . . . . . 34
A.2 Effect of short branches . . . . . . . . . . . . . . . . . . . . . . . . 37
A.3 Model misspecification simulations . . . . . . . . . . . . . . . . . 41
A.4 Bootstrap support values . . . . . . . . . . . . . . . . . . . . . . . 43

**B SatuTe and Tree of Life (ToL)** **45**
B.1 2D ToL - Ribosomal protein alignment . . . . . . . . . . . . . . . . 45
B.1.1 z-score differences between branches . . . . . . . . . . . 45
B.1.2 z-score differences between topologies . . . . . . . . . . . 47
B.2 3D ToL - 16S rRNA alignment . . . . . . . . . . . . . . . . . . . . 48

**C Theoretical foundation of SatuTe** **49**
C.1 Basic properties of the coherence coefficient . . . . . . . . . . . 49
C.2 Testing for Saturation for Any Multiplicity of $\lambda_1$ . . . . . . . . 51
C.3 The Asymptotic Equivalence between the Maximum Likelihood Estimate (MLE) and the Dominant Coherence . . . . . . . . . 52
C.4 Optimal power of the test for saturation . . . . . . . . . . . . . 54

# A SatuTe applied to simulated data

## A.1 Placement of branch $AB$ in the ML-tree

**Protocol for Table S1:** This protocol partially follows the one described for Fig. 2b, see Methods M.4.

| branch length of AB | branch $AB$ connecting 2 external branches | | | branch $AB$ connecting 1 external + 1 internal branch | | |
|---|---|---|---|---|---|---|
| | 100 | 1000 | 10000 | 100 | 1000 | 10000 |
| 0.1 | 0 | 0 | 0 | 10 | 0 | 0 |
| 0.2 | 1 | 0 | 0 | 44 | 0 | 0 |
| 0.3 | 4 | 0 | 0 | 65 | 0 | 0 |
| 0.4 | 10 | 0 | 0 | 120 | 0 | 0 |
| 0.5 | 29 | 0 | 0 | 201 | 0 | 0 |
| 0.8 | 146 | 0 | 0 | 464 | 0 | 0 |
| 1 | 317 | 0 | 0 | 442 | 9 | 0 |
| 1.5 | 601 | 50 | 0 | 323 | 266 | 0 |
| 2 | 736 | 386 | 1 | 238 | 427 | 22 |
| 2.5 | 799 | 616 | 80 | 190 | 330 | 352 |
| 3 | 783 | 697 | 378 | 206 | 279 | 475 |
| 3.5 | 812 | 752 | 556 | 179 | 239 | 399 |
| 4 | 836 | 771 | 657 | 159 | 219 | 317 |
| 5 | 834 | 776 | 699 | 160 | 211 | 291 |
| 7.5 | 844 | 776 | 718 | 145 | 216 | 274 |
| 10 | 828 | 809 | 722 | 162 | 182 | 270 |

**Table S1**: **Placement of the branch** $AB$ **in the ML-tree for the 16-taxon tree.** For each parameter combination of the 16-taxon tree, we simulated 1000 DNA alignments with a sequence length of $100, 1000, 10000$ sites under the JC model. For each alignment, the ML-tree was inferred under the JC model. All ML-trees included a branch that splits the taxa of $\mathbb{T}_A$ and $\mathbb{T}_B$. By examining the splits of the trees, we determined how many $AB$ branches connect either two external branches of the subtrees $\mathbb{T}_A$ and $\mathbb{T}_B$, one external to one internal branch, or two internal branches.

**Protocol for Table S2 and Table S3:** See Methods M.5.

| | **100 bp** | | | | **1 000 bp** | | | | **10 000 bp** | | | |
|---|---|---|---|---|---|---|---|---|---|---|---|---|
| branch length of AB | informative (in %) | accuracy | only AB incorrect | by other reasons | informative (in %) | accuracy | only AB incorrect | by other reasons | informative (in %) | accuracy | only AB incorrect | by other reasons |
| 0.1 | 100 | 632 | 137 | 231 | 100 | 1000 | 0 | 0 | 100 | 1000 | 0 | 0 |
| 0.2 | 100 | 504 | 220 | 276 | 100 | 1000 | 0 | 0 | 100 | 1000 | 0 | 0 |
| 0.3 | 100 | 463 | 257 | 280 | 100 | 999 | 1 | 0 | 100 | 1000 | 0 | 0 |
| 0.4 | 100 | 360 | 365 | 275 | 100 | 1000 | 0 | 0 | 100 | 1000 | 0 | 0 |
| 0.5 | 100 | 290 | 430 | 280 | 100 | 993 | 7 | 0 | 100 | 1000 | 0 | 0 |
| 0.8 | 99.1 | 90 | 592 | 318 | 100 | 935 | 65 | 0 | 100 | 1000 | 0 | 0 |
| 1 | 92.4 | 45 | 629 | 326 | 100 | 825 | 175 | 0 | 100 | 1000 | 0 | 0 |
| 1.5 | 49.7 | 5 | 692 | 303 | 99.9 | 319 | 681 | 0 | 100 | 987 | 13 | 0 |
| 2 | 21.6 | 1 | 683 | 316 | 81.6 | 43 | 957 | 0 | 100 | 744 | 256 | 0 |
| 2.5 | 9.6 | 1 | 659 | 340 | 31.6 | 8 | 992 | 0 | 99.7 | 236 | 764 | 0 |
| 3 | 5.9 | 0 | 656 | 344 | 11.1 | 3 | 997 | 0 | 62.1 | 32 | 968 | 0 |
| 3.5 | 4.3 | 0 | 686 | 314 | 7.4 | 1 | 999 | 0 | 22.8 | 7 | 993 | 0 |
| 4 | 4.6 | 0 | 680 | 320 | 4.3 | 0 | 1000 | 0 | 7.2 | 0 | 1000 | 0 |
| 5 | 3.8 | 0 | 689 | 311 | 3.6 | 0 | 1000 | 0 | 2.7 | 0 | 1000 | 0 |
| 7.5 | 3.5 | 0 | 672 | 328 | 4.8 | 1 | 999 | 0 | 2.5 | 2 | 998 | 0 |
| 10 | 4.3 | 0 | 661 | 339 | 2.7 | 0 | 1000 | 0 | 2.1 | 0 | 1000 | 0 |

**Table S2**: **Impact of saturation on the ML-tree reconstruction for the 16-taxon tree.** For each parameter combination of the 16-taxon tree, we simulated 1000 DNA alignments with a sequence length of $100, 1000, 10000$ sites under the JC model. The ML-tree was inferred for each alignment using the JC model. For the instances of the ML-tree & Bonferroni correction scenario (Fig. 2b, dashed line, green) the table summarises: the fraction of informative instances; the number of correctly reconstructed trees (accuracy); the number of incorrect trees, where solely the branch $AB$ is misplaced between subtrees $\mathbb{T}_A$ and $\mathbb{T}_A$ – both of which are correctly reconstructed as unrooted subtrees; and the number of incorrect trees due to other causes, for each combination of sequence length and branch length.

| branch length of AB | 100 bp | | | | 1 000 bp | | | | 10 000 bp | | | |
|---|---|---|---|---|---|---|---|---|---|---|---|---|
| | informative (in %) | accuracy | only AB incorrect | by other reasons | informative (in %) | accuracy | only AB incorrect | by other reasons | informative (in %) | accuracy | only AB incorrect | by other reasons |
| 0.1 | 100 | 995 | 5 | 0 | 100 | 1000 | 0 | 0 | 100 | 1000 | 0 | 0 |
| 0.2 | 100 | 989 | 11 | 0 | 100 | 1000 | 0 | 0 | 100 | 1000 | 0 | 0 |
| 0.3 | 100 | 995 | 5 | 0 | 100 | 1000 | 0 | 0 | 100 | 1000 | 0 | 0 |
| 0.4 | 100 | 936 | 64 | 0 | 100 | 1000 | 0 | 0 | 100 | 1000 | 0 | 0 |
| 0.5 | 100 | 916 | 84 | 0 | 100 | 1000 | 0 | 0 | 100 | 1000 | 0 | 0 |
| 0.8 | 99 | 686 | 314 | 0 | 100 | 1000 | 0 | 0 | 100 | 1000 | 0 | 0 |
| 1 | 92.6 | 531 | 469 | 0 | 100 | 997 | 3 | 0 | 100 | 1000 | 0 | 0 |
| 1.5 | 53 | 241 | 759 | 0 | 100 | 841 | 159 | 0 | 100 | 1000 | 0 | 0 |
| 2 | 21.6 | 135 | 865 | 0 | 85.8 | 453 | 547 | 0 | 100 | 995 | 5 | 0 |
| 2.5 | 11.9 | 99 | 901 | 0 | 40.3 | 199 | 801 | 0 | 100 | 812 | 188 | 0 |
| 3 | 9.6 | 81 | 919 | 0 | 17.3 | 122 | 878 | 0 | 75.1 | 371 | 629 | 0 |
| 3.5 | 8.3 | 62 | 938 | 0 | 12.3 | 73 | 927 | 0 | 28.5 | 197 | 803 | 0 |
| 4 | 6.8 | 73 | 927 | 0 | 7.3 | 66 | 934 | 0 | 14 | 108 | 892 | 0 |
| 5 | 6 | 64 | 936 | 0 | 5.8 | 46 | 954 | 0 | 4.4 | 78 | 922 | 0 |
| 7.5 | 4.9 | 73 | 927 | 0 | 5 | 49 | 951 | 0 | 4.6 | 76 | 924 | 0 |
| 10 | 5.7 | 60 | 940 | 0 | 5 | 57 | 943 | 0 | 5.1 | 53 | 947 | 0 |

**Table S3**: **Impact of saturation on the ML-tree reconstruction for the 5-taxon tree.** For each parameter combination of the 5-taxon tree, we simulated 1000 DNA alignments with a sequence length of 100, 1000, 10000 sites under the JC model. The ML-tree was inferred for each alignment using the JC model. For the instances of the ML-tree & Bonferroni correction scenario (Fig. 2a, dashed line, green) the table summarises: the fraction of informative instances; the number of correctly reconstructed trees (accuracy); the number of incorrect trees, where solely the branch $AB$ is misplaced between subtrees $\mathbb{T}_A$ and $\mathbb{T}_A$ – both of which are correctly reconstructed as unrooted subtrees; and the number of incorrect trees due to other causes, for each combination of sequence length and branch length.

## A.2 Effect of short branches

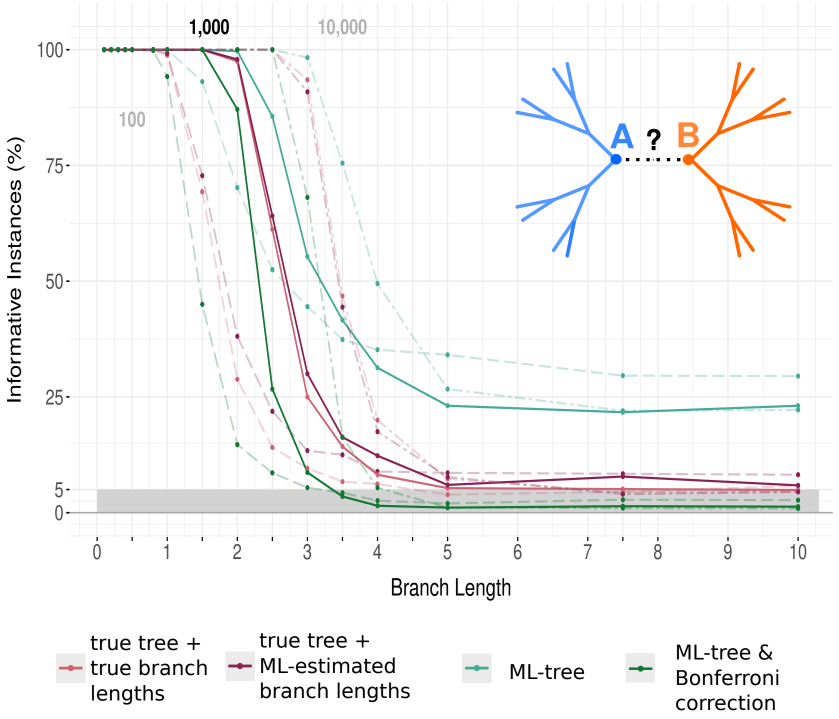


**Fig. S1**: **Effect of short branches on the test for phylogenetic information.** The fraction of phylogenetically informative instances (rejecting the independence of subtrees $\mathbb{T}_A$ and $\mathbb{T}_B$ ) is plotted as a function of the length of branch $AB$ in the true tree in various scenarios: the true tree (pink), the true tree topology with ML-estimated branch lengths (purple), and the inferred ML-tree including branch lengths (blue). The green curves represent the percentage of informative instances after applying the Bonferroni correction to the light green instances. Dashed, solid and alternating dashed lines represent the results for alignment lengths $100, 1000$ and $10000$, respectively. If many of the branch lengths of the tree are close to zero, then the test with Bonferroni correction becomes conservative and the rejection level falls below 5%.

**Protocol for Supplementary Fig. S1:** We closely followed the Protocol for Fig. 2b, see Methods M.4. The only difference was that the internal and external branch lengths were drawn from the distributions (Fig. S2) of internal and external branch lengths, respectively, as recorded in the EvoNAPS database (Reden, 2023, http://evonaps.cibiv.univie.ac.at) with a lower minimum length of $1/n$ (instead of 0.04), where $n$ is the alignment length.

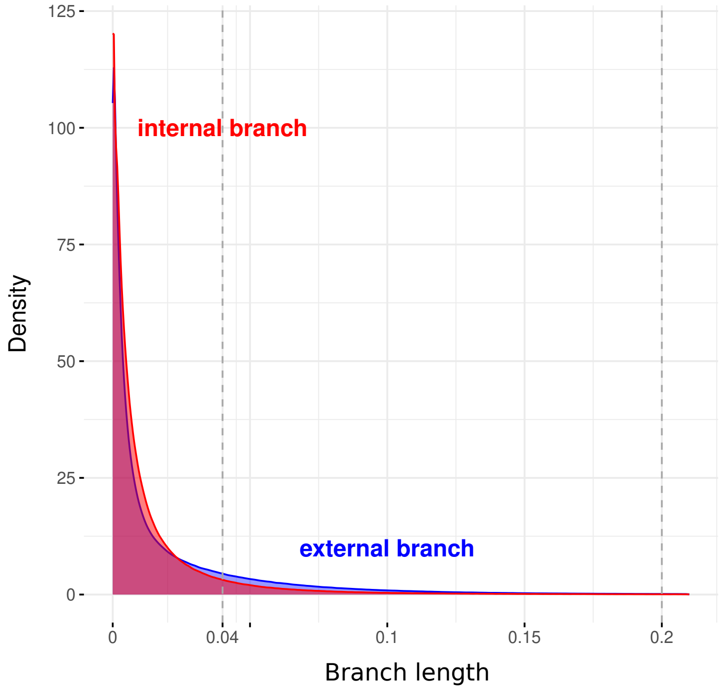


**Fig. S2**: **Empirical branch length distribution from EvoNAPS:** The figure shows the distributions of internal and external branch lengths, respectively, as stored in the EvoNAPS database .

**Protocol for Supplementary Fig. S3 and Table S4:** We closely followed the Protocol for Figure 3, see Methods M.5, using the data from ML-tree & Bonferroni correction scenario presented in Supplementary Fig. S1.

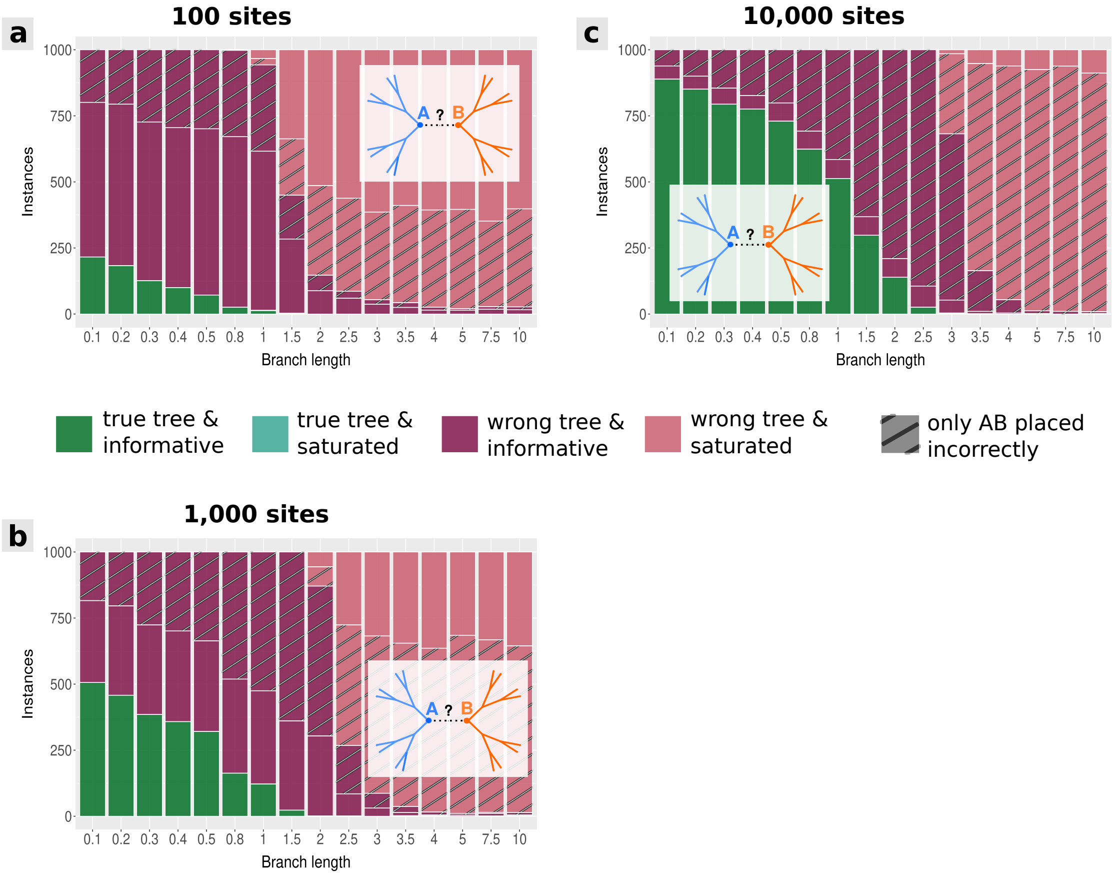


**Fig. S3**: **Saturation and Accuracy.** Further analysis of the ML-tree & Bonferroni correction scenario (dashed line, green) of the 16-taxon tree with short branches in Fig. S1. Bar plots show the fraction of instances based on two conditions: correctly reconstructed trees (green shades) vs. incorrectly reconstructed trees (red shades) and whether branch $AB$ is identified as informative (dark shades) or saturated (light shades). Results are presented for datasets with 100 **(a)**, 1000 **(b)**, and 10,000 **(c)** sites, respectively. Instances of incorrect inference solely due to branch $AB$ being misplaced between the subtrees $\mathbb{T}_A$ and $\mathbb{T}_B$ – both correctly reconstructed as unrooted subtrees – are marked with hatched regions.

| | 100 bp | | | | 1 000 bp | | | | 10 000 bp | | | |
|---|---|---|---|---|---|---|---|---|---|---|---|---|
| branch length of AB | informative (in %) | accuracy | only AB incorrect | by other reasons | informative (in %) | accuracy | only AB incorrect | by other reasons | informative (in %) | accuracy | only AB incorrect | by other reasons |
| 0.1 | 100 | 216 | 199 | 585 | 100 | 506 | 184 | 310 | 100 | 889 | 61 | 50 |
| 0.2 | 100 | 183 | 206 | 611 | 100 | 458 | 204 | 338 | 100 | 851 | 100 | 49 |
| 0.3 | 100 | 126 | 273 | 601 | 100 | 385 | 276 | 339 | 100 | 794 | 145 | 61 |
| 0.4 | 100 | 100 | 295 | 605 | 100 | 358 | 299 | 343 | 100 | 776 | 173 | 51 |
| 0.5 | 100 | 72 | 299 | 629 | 100 | 321 | 336 | 343 | 100 | 730 | 201 | 69 |
| 0.8 | 99.7 | 26 | 328 | 646 | 100 | 163 | 481 | 356 | 100 | 624 | 308 | 68 |
| 1 | 96.5 | 14 | 351 | 635 | 100 | 122 | 525 | 353 | 100 | 513 | 415 | 72 |
| 1.5 | 47.5 | 3 | 379 | 618 | 100 | 23 | 639 | 338 | 100 | 298 | 632 | 70 |
| 2 | 17.4 | 0 | 398 | 602 | 84.5 | 1 | 640 | 359 | 100 | 139 | 790 | 71 |
| 2.5 | 8 | 0 | 378 | 622 | 28.9 | 1 | 639 | 360 | 100 | 26 | 894 | 80 |
| 3 | 4.8 | 0 | 349 | 651 | 6.9 | 0 | 651 | 349 | 67.5 | 4 | 932 | 64 |
| 3.5 | 3.1 | 0 | 386 | 614 | 3 | 2 | 641 | 357 | 17.5 | 1 | 938 | 61 |
| 4 | 2.7 | 0 | 379 | 621 | 1.8 | 1 | 631 | 368 | 4.3 | 0 | 935 | 65 |
| 5 | 2.7 | 0 | 382 | 618 | 1.5 | 0 | 681 | 319 | 1.2 | 1 | 923 | 76 |
| 7.5 | 2.8 | 0 | 334 | 666 | 1.2 | 1 | 657 | 342 | 0.6 | 0 | 938 | 62 |
| 10 | 1.6 | 0 | 382 | 618 | 1 | 1 | 640 | 359 | 0.9 | 0 | 910 | 90 |

**Table S4**: **Impact of saturation on the ML-tree reconstruction for the 16-taxon tree with short branches.** For each parameter combination of the 16-taxon tree, we simulated 1000 DNA alignments with a sequence length of $100, 1000, 10000$ sites under the JC model. The ML-tree was inferred for each alignment using the JC model. For the instances of the ML-tree & Bonferroni correction scenario (Fig. S1, dashed line, green) the table summarises: the fraction of informative instances; the number of correctly reconstructed trees (accuracy); the number of incorrect trees, where solely the branch $AB$ is misplaced between subtrees $\mathbb{T}_A$ and $\mathbb{T}_A$ – both of which are correctly reconstructed as unrooted subtrees; and the number of incorrect trees due to other causes, for each combination of sequence length and branch length.

## A.3 Model misspecification simulations

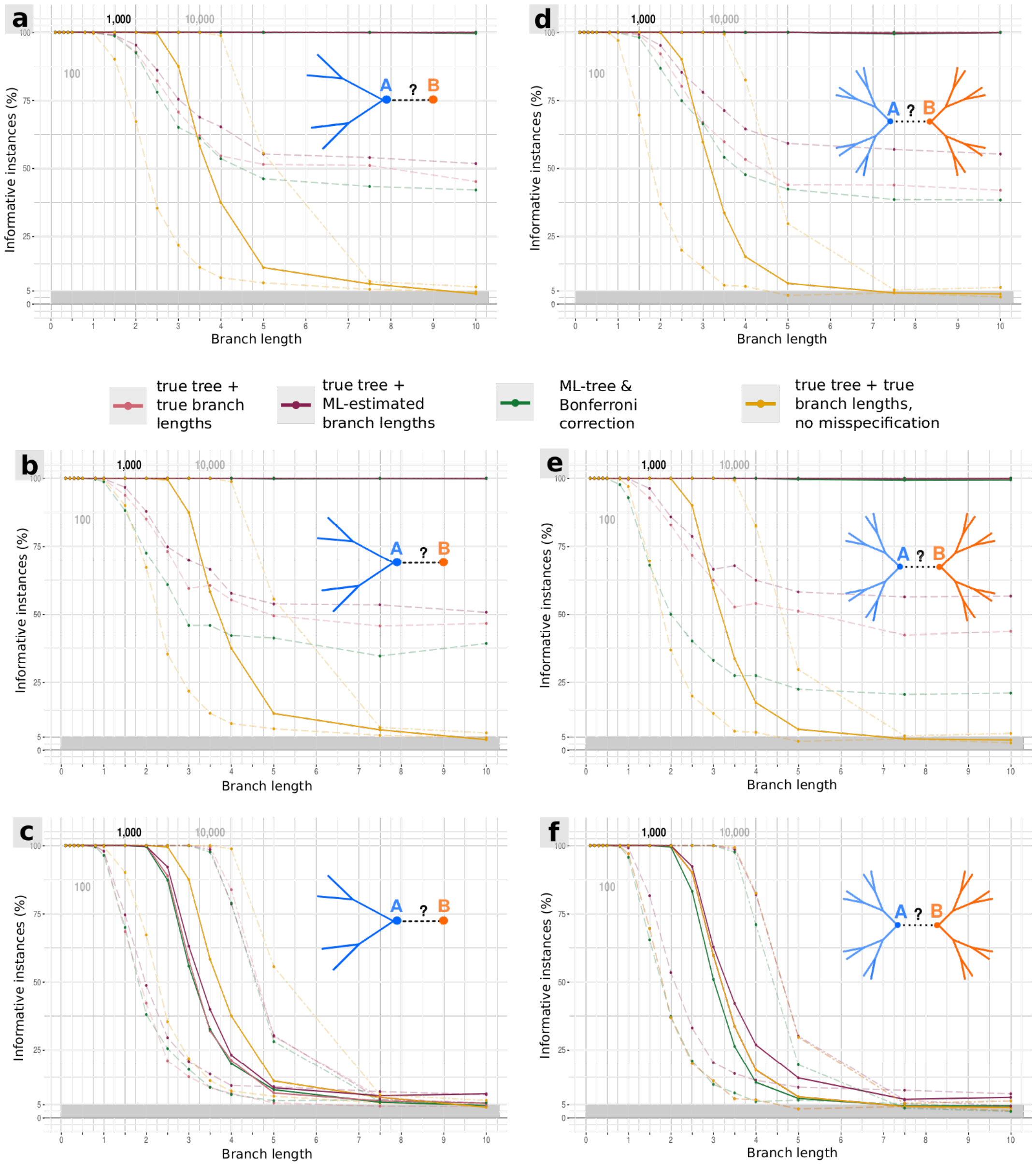


**Fig. S4**: **Performance of SatuTe in presence of model misspecification.** The plot shows the fraction of informative instances when testing the branch $AB$ in absence of model misspecification (true-tree, gold) and in various scenarios with model misspecification: true-tree (pink), the true-tree topology with ML-estimated branch lengths (purple), and the inferred ML-tree including branch lengths with Bonferroni correction (green). In all cases the alignments were simulated with a GTR model, while reconstruction and testing under model misspecification was performed using the JC **(a,d)**, K2P **(b,e)** or F81 **(c,f)** models. The branches tested were **(a-c)** the external branch $AB$ of a 5-taxon tree and **(d-f)** the central branch $AB$ of a balanced 16-taxon tree. Dashed, solid and alternating dashed lines represent the results for alignments with 100, 1000 and 10000 number of sites, respectively.

**Protocol for Supplementary Fig. S4:** This protocol is similar to the protocol in Methods M.4.

We consider the same simulation trees as described (for details see M.4. and Table 1). For each parameter combination, we simulated 1000 DNA alignments with sequence length $n = 100, 1000, 10000$ sites using Seq-Gen (v1.3.4; Rambaut and Grassly, 1997) under a GTR model with rates $AC = 0.6676$, $AG = 3.7807$, $AT = 4.2833$, $CG = 0.5354$, $CT = 0.8718$ and $GT = 1.0$ and nucleotide frequencies $\pi_A = 0.125$, $\pi_C = 0.436$, $\pi_G = 0.191$ and $\pi_T = 0.245$, as estimated for the best-fit model for the dataset PF06346 stored in the EvoNAPS database (Reden, 2023, http://evonaps.cibiv.univie.ac.at). Each alignment was evaluated using IQ-Tree2 (Minh et al., 2020) and SatuTe with a significance level of $\alpha = 0.05$ while specifying JC **(a,d)**, K2P **(b,e)** and F81 **(c,f)** as models of substitution instead of GTR. Missing model parameters are estimated from the alignments.

The fraction of phylogenetically informative alignments (rejecting the independence of subtrees $\mathbb{T}_A$ and $\mathbb{T}_B$) is plotted as a function of the branch length $AB$ in the simulation tree for four different analyses:

1. Applying the test for phylogenetic information on the true simulation tree with fixed true branch lengths (gold), assuming the GTR model of the simulation for each alignment. (First scenario with good specification)
2. Applying the test for phylogenetic information on the true simulation tree with fixed true branch lengths (pink), assuming the JC **(a,d)**, K2P **(b,e)** and F81 **(c,f)** model for each alignment. (First scenario with model misspecification)
3. Applying the test for phylogenetic information on the true tree topology with ML-estimated branch lengths (purple), assuming the JC **(a,d)**, K2P **(b,e)** and F81 **(c,f)** model for each alignment. (Second scenario with model misspecification)
4. Applying the test for phylogenetic information with the Bonferroni correction $\alpha_{adjust}$ (see Eq. (11)) on the inferred ML-tree with estimated branch lengths (green), assuming the JC **(a,d)**, K2P **(b,e)** and F81 **(c,f)** model for each alignment. (Fourth scenario with model misspecification)

Note that, for the GTR model, the second largest eigenvalue has a multiplicity of 1. The JC model **(a,d)** and the F81 model **(c,f)** have a unique non-zero eigenvalue with multiplicity 3. For the K2P model **(b,e)**, the second largest eigenvalue has multiplicity 1 or 2, depending on the transition/transversion ratio. The details of the test are as described in Methods M.2.

| | Fig. S4a,b,c | Fig. S4d,e,f |
|---|---|---|
| **simulation** | | |
| **tree:** | 5-taxon tree | 16-taxon tree |
| **branch AB:** | external, see Fig. 2a | internal, see Fig. 2b |
| **branches in $\mathbb{T}_A$ & $\mathbb{T}_B$:** | all 0.2 | drawn from EvoNAPS |
| **with length range:** | NA | 0.04 to 0.2 |
| **substitution model:** | GTR model | GTR model |
| **evaluation** | | |
| **substitution model:** | (a) JC model<br>(b) K2P model<br>(c) F81 model | (d)JC model<br>(e) K2P model<br>(f) F81 model |
| **Bonferroni correction:** | $\alpha_{adjust} = 0.05/4$ | $\alpha_{adjust} = 0.05/64$ |

**Table S5**: **SatuTe in simulations with model misspecifications.**

Branch lengths of the subtrees $\mathbb{T}_A$ and $\mathbb{T}_B$ in the 16-taxon trees were drawn between 0.04 and 0.2 from the distributions of internal and external branch lengths, respectively (see Supplementary Fig. S2), as stored in the EvoNAPS database (Reden, 2023, http://evonaps.cibiv.univie.ac.at).

Each datapoint in Fig. S4 has been computed from an independent set of 1000 alignments. In the rare cases where the inferred ML-tree did not recover the split induced by branch $AB$ (i.e., the taxa of $\mathbb{T}_A$ did not split from the taxa of $\mathbb{T}_B$), the ML-tree was discarded. This only occurred for simulated alignments with $n = 100$ and $AB$ branch length 0.1 or 0.2 (totalling $4 \times 1000$ alignments). The maximum number of discarded ML-trees occurred under the F81 model (Fig. S4f), where 1.6% (64 out of $4 \times 1000$) of ML-trees where discarded.

## A.4 Bootstrap support values

**Protocol for Table S6:** This protocol partially follows the one described for Fig. 2b, see Methods M.4.
The considered simulation tree is the 16-taxon tree shown in Fig. 2b. Our focus is on the internal branch $AB$, which connects the root $A$ of the balanced 8-taxon subtree $\mathbb{T}_A$ to the root $B$ of thw balanced 8-taxon subtree $\mathbb{T}_B$. All branch lengths in $\mathbb{T}_A$ and $\mathbb{T}_\mathbb{B}$ were drawn between 0.04 and 0.2 from the distributions (see Supplementary Fig. S2) of internal and external branch lengths, respectively, as collected in the EvoNAPS database (Reden, 2023, http://evonaps.cibiv.univie.ac.at) . As before, the $AB$-branch length was varied among $\{0.1, 0.2, 0.3, 0.4, 0.5, 0.8, 1.0, 1.5, 2.0, 2.5, 3.0, 3.5, 4.0, 5.0, 7.5, 10.0\}$. For each parameter combination, we simulated 1000 DNA alignments with sequence length of 100 sites using Seq-Gen (v1.3.4; Rambaut and Grassly, 1997) under the JC model. For each alignment, we conducted a bootstrap analysis with 100 replicates using IQ-Tree2 (Minh et al., 2020). For each of

the 1000 considered ML-tree, we determined if a branch exists that splits the taxa of $\mathbb{T}_A$ and $\mathbb{T}_B$ (i.e., $AB$ split contained). For the ML-trees containing this split, the bootstrap support value of branch $AB$ was computed using 100 resamplings, allowing us to calculate the average and minimum BS values. For comparison, we include the fraction of informative instances of Fig. 2b for an alignment length of 100 sites in the ML-tree & Bonferroni correction scenario (dashed line, green). See Protocol for Fig. 2b (Methods M.4).

Table S6 only shows the results for simulated alignments with length $n = 100$. For longer alignments (1000, 10000) the subtrees were always separated and the BS values were always 100%, independently of the $AB$ branch length.

| simulation branch length of $AB$ | $AB$ split contained | Average BS value | Minimum BS value | Informative instances (%) |
|---|---|---|---|---|
| 0.1 | 993 | 93.32 | 14 | 100 |
| 0.2 | 999 | 99.19 | 74 | 100 |
| 0.3 | 1000 | 99.86 | 87 | 100 |
| 0.4 | 1000 | 99.95 | 93 | 100 |
| 0.5 | 1000 | 99.99 | 97 | 100 |
| 0.8 | 1000 | 100 | 100 | 99.1 |
| 1 | 1000 | 100 | 100 | 92.4 |
| 1.5 | 1000 | 100 | 100 | 49.7 |
| 2 | 1000 | 100 | 100 | 21.6 |
| 2.5 | 1000 | 100 | 100 | 9.6 |
| 3 | 1000 | 100 | 100 | 5.9 |
| 3.5 | 1000 | 100 | 100 | 4.3 |
| 4 | 1000 | 100 | 100 | 4.6 |
| 5 | 1000 | 100 | 100 | 3.8 |
| 7.5 | 1000 | 100 | 100 | 3.5 |
| 10 | 1000 | 100 | 100 | 4.3 |

**Table S6**: **Bootstrap support (BS) values of the simulations for the 16-taxon tree.** For each branch length of $AB$ in the simulation tree as shown in Fig. 2b, 1000 DNA alignments with sequence length of 100 sites were simulated under JC model. For each alignment, we conducted a bootstrap analysis with 100 replicates. The table summarises the number of inferred ML-trees containing a branch that splits the taxa of $\mathbb{T}_A$ and $\mathbb{T}_B$ ($AB$ split contained), along with the average and minimum bootstrap support values over all 1000 analyses. For comparison, we include the fraction of informative instances of Fig. 2b for an alignment length of 100 sites in the scenario ML-tree & Bonferroni correction (dashed line, green).

# B SatuTe and Tree of Life (ToL)

We apply SatuTe to real data by analysing the Tree of Life (ToL) for ribosomal protein and rRNA alignments. For this purpose, we are examining the work of Hug et al. (2016).

Using SatuTe, we can perform a wide range of per-alignment-region analyses considering windows, different proteins or rRNA regions as presented in the main text. Additionally to the insights of the phylogenetic signals for this regions, the resulting z-scores enable us to make comparisons, for instance, between branches or even different topologies.

## B.1 2D ToL - Ribosomal protein alignment

### B.1.1 z-score differences between branches

**Protocol for Table S7 and Fig. S5**: To compare the phylogenetic signal in protein sequences between branches leading to Eukaryota and baker's yeast (S. cerevisiae), we performed the test according to Methods M.3 using LG+Γ4 model and considering the different proteins. For the comparison, we calculate the difference in per-protein z-scores. The aggregated results are presented in Table S7 and Fig. S5.

| protein | z-score Eukaryota | z-score yeast | Δ z-score (Euk.- Yeast) |
|---|---|---|---|
| L2 | 11.68 | 12.85 | -1.17 |
| L3 | 11.62 | 11.38 | 0.24 |
| L4 | 11.06 | 12.23 | -1.17 |
| L5 | 12.31 | 12.54 | -0.23 |
| L6 | 10.34 | 10.49 | -0.15 |
| L14 | 9.96 | 10.47 | -0.51 |
| L15 | 7.72 | 8.88 | -1.16 |
| L16 | 6.35 | 11.30 | -4.95 |
| L18 | 8.23 | 11.82 | -3.59 |
| L22 | 8.28 | 11.70 | -3.42 |
| L24 | 7.77 | 7.55 | 0.22 |
| S3 | 10.85 | 12.81 | -1.96 |
| S8 | 10.03 | 10.48 | -0.45 |
| S10 | 8.91 | 9.15 | -0.24 |
| S17 | 7.92 | 7.23 | 0.69 |
| S19 | 8.35 | 8.49 | -0.14 |

**Table S7**: **Per-protein z-score comparison between two branches of the 2D ToL.** For each ribosomal protein, the table summarises the the z-score of the branch leading to Eukaryota and yeast in the 2D ToL of Fig. 4a, along with their differences.

All z-scores are greater than the critical value $z_\alpha$ or $z_{\alpha_{adjusted}}$ for significance $\alpha = 0.01$. Considering the per-protein z-score difference between Eukaryota

and yeast, Supplementary Fig. S5 shows that most of differences in z-score are small or close to zero, with the notable exceptions of L16, L18 and L22, where the Eukaryota branch shows comparatively less phylogenetic signal.

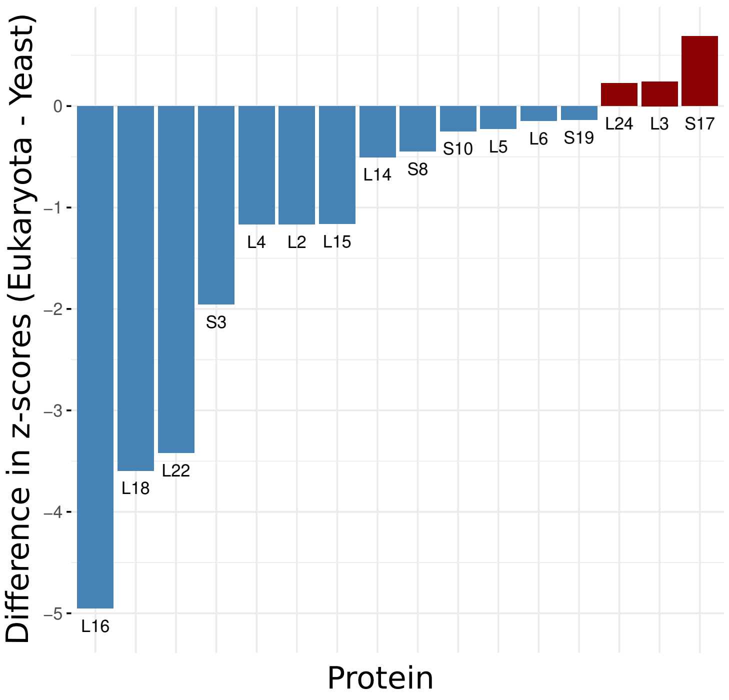


**Fig. S5**: **Per-protein z-score comparison between the branches leading to Eukaryota and Yeast.** For each ribosomal protein, the z-score differences between the branch leading to Eukaryotes and the branch leading to Yeast in the 2D ToL of Fig. 4a. are presented in ascending order.

### B.1.2 z-score differences between topologies

In addition to the branch comparison, z-score differences allow us to determine whether each protein supports the 2D ToL or the 3D ToL.

**Protocol for Table S8:** See the Protocol for Fig. 5.

| protein | alignment length | z-score 2D ToL | z-score rearranged 3D ToL | Δ z-score (2D-3D) |
|---|---|---|---|---|
| L2 | 317 | 11.68 | 11.65 | 0.03 |
| L3 | 228 | 11.62 | 11.07 | 0.55 |
| L4 | 245 | 11.06 | 11.02 | 0.04 |
| L5 | 164 | 12.31 | 12.21 | 0.10 |
| L6 | 220 | 10.34 | 10.27 | 0.07 |
| L14 | 125 | 9.96 | 9.01 | 0.95 |
| L15 | 122 | 7.72 | 8.75 | -1.03 |
| L16 | 153 | 6.35 | 6.30 | 0.05 |
| L18 | 141 | 8.23 | 8.11 | 0.12 |
| L22 | 174 | 8.28 | 8.69 | -0.41 |
| L24 | 81 | 7.77 | 7.68 | 0.09 |
| S3 | 216 | 10.85 | 10.51 | 0.34 |
| S8 | 141 | 10.03 | 9.77 | 0.26 |
| S10 | 104 | 8.91 | 9.04 | -0.13 |
| S17 | 74 | 7.92 | 7.37 | 0.55 |
| S19 | 91 | 8.35 | 8.01 | 0.34 |

**Table S8**: **Per-protein z-score comparison to a rearranged topology:** For each ribosomal protein, the table summarises the z-scores of the branch leading to Eukaryota in the 2D ToL of Fig. 4a and the 3D-rearranged tree of Fig. 5a, along with their differences.

## B.2 3D ToL - 16S rRNA alignment

### z-score differences between topologies

For comparison, we determine the z-score differences of different rRNA regions for the 2D or the 3D ToL.

**Protocol for Table S9:** We divided the 16S rRNA gene alignment into nine distinct rRNA-regions, as detailed in Table S9. See the Protocol for Fig. 5.

| region | site range | z-score 3D ToL | z-score rearranged 2D ToL | $\Delta$ z-score (2D-3D) |
|---|---|---|---|---|
| R1 | 0-114 | 4.57 | 5.08 | 0.51 |
| R2 | 115-431 | 6.13 | 5.94 | -0.19 |
| R3 | 432-621 | 3.63 | 3.45 | -0.17 |
| R4 | 622-1066 | 6.81 | 6.83 | 0.03 |
| R5 | 1067-1221 | 8.99 | 8.93 | -0.06 |
| R6 | 1222-1394 | 8.55 | 8.31 | -0.23 |
| R7 | 1395-1565 | 8.93 | 8.35 | -0.59 |
| R8 | 1566-1765 | 12.44 | 12.33 | -0.12 |
| R9 | 1766-1946 | 9.33 | 9.65 | 0.33 |

**Table S9**: **Per-region z-score comparison to a rearranged topology** For each rRNA region, the table summarises the z-scores of the branch leading to Eukaryota in the ML-reconstructed 3D ToL (Fig. 4d) and the rearranged 2D ToL (Fig. 5c), along with their differences.

# C Theoretical foundation of SatuTe

## C.1 Basic properties of the coherence coefficient

As inferred from Eq. (3), for pattern $\partial$ the coherence coefficient corresponding to eigenvalue $\lambda_i$ is defined as

$$C_i^{\partial} := \langle \frac{\boldsymbol{L}(\partial A)}{\mathbb{P}(\partial A)}, \boldsymbol{h_i} \rangle \langle \frac{\boldsymbol{L}(\partial B)}{\mathbb{P}(\partial B)}, \boldsymbol{h_i} \rangle. \tag{C1}$$

Let us restate the spectral decomposition of Eq. (2). Due to reversibility, the transition matrix $\boldsymbol{P}(t) = e^{Qt}$ decomposes as

$$\boldsymbol{P}(t) = \boldsymbol{1}\boldsymbol{\pi}^T + \sum_{i=1}^{3} \boldsymbol{v_i}\boldsymbol{h_i}^T e^{\lambda_i t}, \tag{C2}$$

where $\lambda_0 = 0 > \lambda_1 \geq \lambda_2 \geq \lambda_3$ are the eigenvalues of the rate matrix and $\boldsymbol{h_i}, \boldsymbol{v_i}$ are the corresponding left and right eigenvectors. Note that the right eigenvalues form an orthonormal basis with respect to $\boldsymbol{\pi}$-inner product $\langle \boldsymbol{v}, \boldsymbol{w} \rangle_{\boldsymbol{\pi}} = \boldsymbol{v}^T \cdot \mathrm{Diag}(\boldsymbol{\pi}) \cdot \boldsymbol{w}$ and $\boldsymbol{h}_k = \mathrm{Diag}(\boldsymbol{\pi})\boldsymbol{v}_k$ (Levin and Peres, 2017).

To simplify the computation of the expected value of the coherence coefficient, in Prop. C.1 we consider each scalar product of Eq. (C1) independently.

**Proposition C.1.** *Assuming stationarity, for each pattern $\partial A$ observed at the tips of subtree $\mathbb{T}_A$, consider the likelihood vector $\boldsymbol{L}(\partial A)$ and the probability $\mathbb{P}(\partial A) = \langle \boldsymbol{\pi}, \boldsymbol{L}(\partial A) \rangle$. The following holds.*

*a) If $\boldsymbol{1}$ denotes the column vector of 1's, then*

$$\mathbb{E}\Big[\frac{\boldsymbol{L}(\partial A)}{\mathbb{P}(\partial A)}\Big] = \boldsymbol{1}.$$

*b) If vector $\boldsymbol{h_i}$ is orthogonal to $\boldsymbol{1}$, then it holds that*

$$\mathbb{E}\Big[\langle \frac{\boldsymbol{L}(\partial A)}{\mathbb{P}(\partial A)}, \boldsymbol{h_i} \rangle\Big] = 0.$$

*Proof*

a) It is enough to show that

$$\sum_{\partial A} \boldsymbol{L}(\partial A) = \boldsymbol{1}. \tag{C3}$$

Consider one nucleotide $i$. Focusing on the $i$th entry of $\boldsymbol{L}(\partial A)$, we see that

$$\sum_{\partial A} \mathbb{P}(\partial A \mid i \text{ at node } A) = 1$$

because we are summing over all possible outcomes $\partial A$, as desired.

b) We just need to use linearity and item $a$). Indeed,

$$\mathbb{E}\Big[\langle\frac{\boldsymbol{L}(\partial A)}{\mathbb{P}(\partial A)},\boldsymbol{h_i}\rangle\Big]=\langle\mathbb{E}\Big[\frac{\boldsymbol{L}(\partial A)}{\mathbb{P}(\partial A)}\Big],\boldsymbol{h_i}\rangle=\langle\boldsymbol{1},\boldsymbol{h_i}\rangle=0, \tag{C4}$$

where in last equality we used the fact that $\boldsymbol{h_i}$ and $\boldsymbol{1}$ are orthogonal, as it is the case in the spectral decomposition of Eq. (C2).

□

Recall that Eq. (4) implies that, when the length of branch $AB$ grows, the subpatterns $\partial A$ and $\partial B$ become independent. Using Prop. C.1, we can easily compute the expected value $\mathbb{E}[C_i^{\partial}]$ under the assumption of subtree independence as follows.

**Proposition C.2.** *In a phylogenetic tree $\mathbb{T}$, consider a branch $AB$ with length $t\to\infty$. Assuming stationarity and reversibility, for any $i\in\{1,2,3\}$ it holds that*

$$\mathbb{E}[C_i^{\partial}]=0.$$

*Proof* Since subpatterns $\partial A$ and $\partial B$ are sampled independently, also $\langle\boldsymbol{L}(\partial A)/\mathbb{P}(\partial A),\boldsymbol{h_i}\rangle$ and $\langle\boldsymbol{L}(\partial B)/\mathbb{P}(\partial B),\boldsymbol{h_i}\rangle$ are independent. The expectation is multiplicative under independence, and thus we have

$$\mathbb{E}[C_i^{\partial}]=\mathbb{E}\Big[\langle\frac{\boldsymbol{L}(\partial A)}{\mathbb{P}(\partial A)},\boldsymbol{h_i}\rangle\langle\frac{\boldsymbol{L}(\partial B)}{\mathbb{P}(\partial B)},\boldsymbol{h_i}\rangle\Big]= \tag{C5}$$

$$=\mathbb{E}\Big[\langle\frac{\boldsymbol{L}(\partial A)}{\mathbb{P}(\partial A)},\boldsymbol{h_i}\rangle\Big]\mathbb{E}\Big[\langle\frac{\boldsymbol{L}(\partial B)}{\mathbb{P}(\partial B)},\boldsymbol{h_i}\rangle\Big]=0, \tag{C6}$$

where we used Prop. C.1b.

□

As described in Methods M.1, for any $i,j\in\{1,2,3\}$ we define the covariance

$$\sigma^2_{A,i,j}:=\mathbb{E}\Big[\langle\frac{\boldsymbol{L}(\partial A)}{\mathbb{P}(\partial A)},\boldsymbol{h_i}\rangle\langle\frac{\boldsymbol{L}(\partial A)}{\mathbb{P}(\partial A)},\boldsymbol{h_j}\rangle\Big]. \tag{C7}$$

This is indeed the covariance between the two scalar products due to Prop. C.1b. As claimed in Methods M.1, in the following proposition we prove that $\sigma^2_{A,i,j}$ greatly simplifies if node $A$ is external.

**Proposition C.3.** *Assuming stationarity and reversibility, if node $A$ is external (or equivalently, if subtree $\mathbb{T}_A$ is composed by a single sequence), then*

$$\sigma^2_{A,i,j}=\begin{cases}1, & \text{if } i=j.\\ 0, & \text{if } i\neq j.\end{cases}$$

*Proof* In the eigendecomposition of Eq. (C2), we know that $\mathrm{Diag}(\boldsymbol{\pi})\boldsymbol{v_i}=\boldsymbol{h_i}$, while $\langle\boldsymbol{v_i},\boldsymbol{h_j}\rangle=1$ and $\langle\boldsymbol{v_i},\boldsymbol{h_j}\rangle=0$ if $i\neq j$. The patterns of a single sequence are the possible letters $x$ it is composed of. Moreover, due to stationarity, we know that

pattern $\partial A = x$ satisfies $\mathbb{P}(\partial A) = \pi_x$, where $x$ is a nucleotide. Note also that, if $\partial A = x$, then $\boldsymbol{L}(\partial A)$ is the vector of 0's, except 1 at the $x$th entry. All in all, we have

$$\sigma^2_{A,i,i} = \sum_x \mathbb{P}(x)(\frac{1}{\pi_x} h_i^x)^2 = \sum_x \pi_x(\frac{1}{\pi_x} h_i^x)^2 = \sum_x v_i^x h_i^x = \langle \boldsymbol{h_i}, \boldsymbol{v_i} \rangle = 1. \tag{C8}$$

Similarly, if $i \neq j$ we have

$$\sigma^2_{A,i,j} = \sum_x \mathbb{P}(x)(\frac{1}{\pi_x} h_i^x)(\frac{1}{\pi_x} h_j^x) = \sum_x \pi_x(\frac{1}{\pi_x} h_i^x)(\frac{1}{\pi_x} h_j^x) = \langle \boldsymbol{h_i}, \boldsymbol{v_j} \rangle = 0. \tag{C9}$$

□

## C.2 Testing for Saturation for Any Multiplicity of $\boldsymbol{\lambda_1}$

Any linear combination of the coherence coefficients them can be used to test for saturation. However, we want the most powerful test among the candidates. As we prove in Appendix C.4, optimal power is achieved asymptotically by the test that uses the coherence coefficient introduced in Eq. (7), namely

$$\hat{C}_1 := \sum_{k \in \{1, \cdots, D\}} \hat{C}_{1,k}, \tag{C10}$$

where eigenvalue $\lambda_1$ has multiplicity $D$ and $\lambda_1 = \cdots = \lambda_D$. Under the null hypothesis of subtree independence, the expectation and variance of $\hat{C}_1$ can be computed as described in the following proposition.

**Proposition C.4.** *In a phylogenetic tree $\mathbb{T}$ consider a branch AB with length $t^*$ and assume stationarity and reversibility. If $t^* \to \infty$, then the coherence coefficient $\hat{C}_1$ satisfies*

$$\mathbb{E}[\hat{C}_1] = 0, \tag{C11}$$

$$\mathrm{Var}[\hat{C}_1] = \frac{1}{n} \sum_{i,j \in \{1, \cdots, D\}} \sigma^2_{A,i,j} \sigma^2_{B,i,j}. \tag{C12}$$

*In particular, if node A is external, then*

$$\mathrm{Var}[\hat{C}_1] = \frac{1}{n} \sum_{i \in \{1, \cdots, D\}} \sigma^2_{B,i,i}, \tag{C13}$$

*and if both nodes A and B are external, then*

$$\mathrm{Var}[\hat{C}_1] = \frac{D}{n}. \tag{C14}$$

*Proof* Since the expectation of a sum is the sum of expectations, $\mathbb{E}[C^{\partial}_{1,i}] = 0$ (Prop. C.2) implies that $\mathbb{E}[\hat{C}_1] = 0$.

Regarding the variance, we can sum up the variance of each independent site, giving $\mathrm{Var}[\hat{C}_1] = 1/n\mathrm{Var}[\sum_{i\in\{1,\cdots,D\}} C^{\partial}_{1,i}]$. Since $\mathbb{E}[C^{\partial}_{1,i}] = 0$ (Prop. C.2), we have

$$\mathrm{Var}[\sum_{i\in\{1,\cdots,D\}} C^{\partial}_{1,i}] = \mathbb{E}[(\sum_{i\in\{1,\cdots,D\}} C^{\partial}_{1,i})^2]. \tag{C15}$$

After expanding the squared sum $(\sum_{i\in\{1,\cdots,D\}} C^{\partial}_{1,i})^2$, since the expectation is multiplicative under independence, a summand $C^{\partial}_{1,i}C^{\partial}_{1,j}$ satisfies

$$\begin{aligned}&\mathbb{E}[C^{\partial}_{1,i}C^{\partial}_{1,j}] = \qquad\qquad (\mathrm{C16})\\ =&\mathbb{E}\Big[\langle\frac{\boldsymbol{L}(\partial A)}{\mathbb{P}(\partial A)},\boldsymbol{h_i}\rangle\langle\frac{\boldsymbol{L}(\partial B)}{\mathbb{P}(\partial B)},\boldsymbol{h_i}\rangle\langle\frac{\boldsymbol{L}(\partial A)}{\mathbb{P}(\partial A)},\boldsymbol{h_j}\rangle\langle\frac{\boldsymbol{L}(\partial B)}{\mathbb{P}(\partial B)},\boldsymbol{h_j}\rangle\Big] =\\ =&\mathbb{E}\Big[\langle\frac{\boldsymbol{L}(\partial A)}{\mathbb{P}(\partial A)},\boldsymbol{h_i}\rangle\langle\frac{\boldsymbol{L}(\partial A)}{\mathbb{P}(\partial A)},\boldsymbol{h_j}\rangle\Big]\mathbb{E}\Big[\langle\frac{\boldsymbol{L}(\partial B)}{\mathbb{P}(\partial B)},\boldsymbol{h_i}\rangle\langle\frac{\boldsymbol{L}(\partial B)}{\mathbb{P}(\partial B)},\boldsymbol{h_j}\rangle\Big] =\\ =&\sigma^2_{A,i,j}\sigma^2_{B,i,j}.\end{aligned}$$

Summing for all $i,j \in \{1,\cdots,D\}$ we get the desired equality. The particular case when nodes $A$ and/or $B$ are external follows from Prop. C.3.

□

Prop. C.4 leads to a one-sided $z$-test based on the coherence coefficient $\hat{C}_1$. The null hypothesis of independence is rejected with significance $\alpha$ if

$$\hat{C}_1 > z_\alpha\sqrt{\widehat{\mathrm{Var}}[\hat{C}_1]} = z_\alpha\frac{\sqrt{\sum_{i,j\in\{1,\cdots,D\}}\hat{\sigma}^2_{A,i,j}\hat{\sigma}^2_{B,i,j}.}}{\sqrt{n}}, \tag{C17}$$

where $z_\alpha$ satisfies $\mathbb{P}(Z > z_\alpha) = \alpha$, while $\hat{\sigma}^2_{A,i,j}$ is the sample estimate of $\sigma^2_{A,i,j}$ introduced in Eq. (8). If node $A$ is external, then Eq. C17 is simplified due to Prop. C.3 as

$$\hat{C}_1 > z_\alpha\frac{\sqrt{\sum_{i\in\{1,\cdots,D\}}\hat{\sigma}^2_{B,i,i}}}{\sqrt{n}}. \tag{C18}$$

## C.3 The Asymptotic Equivalence between the Maximum Likelihood Estimate (MLE) and the Dominant Coherence

There is a close relationship between the coherence coefficient and the log-likelihood of a branch. To describe this relationship, consider branch $AB$ of tree $\mathbb{T}$. Given an alignment where pattern $\partial$ is observed $n_\partial$ times, the log-likelihood $f(t)$ of branch $AB$ having length $t$ is

$$f(t) = \sum_{\partial} n_\partial \log\left(\mathbb{P}(\partial \mid t)\right). \tag{C19}$$

If we define the log-likelihood of independence as

$$f_\infty := \sum_\partial n_\partial \Big( \log \mathbb{P}(\partial A) + \log \mathbb{P}(\partial B) \Big), \tag{C20}$$

then Eq. (C19) can be rewritten as

$$f(t) - f_\infty = \sum_\partial n_\partial \log \big( \frac{\mathbb{P}(\partial \mid t)}{\mathbb{P}(\partial A)\mathbb{P}(\partial B)} \big). \tag{C21}$$

Using Eq. (4), last equation becomes

$$f(t) - f_\infty = \sum_\partial n_\partial \log \Big( 1 + \sum_{i \in \{1,2,3\}} e^{\lambda_i t} C_i^\partial \Big). \tag{C22}$$

In Prop. C.5, we formalise the relationship between the coherence coefficient and the log-likelihood $f(t)$.

**Proposition C.5.** *Assuming stationarity and reversibility, if rate matrix $Q$ has eigenvalues $0 > \lambda_1 \geq \lambda_2 \geq \lambda_3$ and $\lambda_1$ has multiplicity $D$, then define the coherence coefficient*

$$\hat{C}_1 := \sum_{i \in \{1,\cdots,D\}} \hat{C}_{1,i}.$$

*If $\hat{C}_1 \neq 0$, then*

$$f(t) - f_\infty \sim \hat{C}_1 n e^{\lambda_1 t}.$$

*Proof*
In Equation C21 it is clear that $f(t) - f_\infty$ tends to zero as $t \to \infty$, since $\log(1) = 0$. Now, using L'Hôpital's rule, it is enough to show that the derivative $f'(t)$ satisfies $f'(t) \sim \hat{C}_1 n \lambda_1 e^{\lambda_1 t}$. In the expression

$$[\log \Big( 1 + \sum_{i \in \{1,2,3\}} e^{\lambda_i t} C_i^\partial \Big)]' = \frac{\sum_{i \in \{1,2,3\}} \lambda_i e^{\lambda_i t} C_i^\partial}{1 + \sum_{i \in \{1,2,3\}} e^{\lambda_i t} C_i^\partial},$$

the denominator is asymptotically equivalent to 1. On the other hand, if $\sum_{i \in \{1,\cdots,D\}} C_i^\partial \neq 0$, then the numerator is equivalent to $\lambda_1 e^{\lambda_1 t} \sum_{i \in \{1,\cdots,D\}} C_i^\partial$, and $o(e^{\lambda_1 t})$ otherwise. Due to the assumption $\hat{C}_1 \neq 0$, we can sum up the equivalencies, giving

$$f'(t) \sim \sum_\partial n_\partial \Big( \lambda_1 e^{\lambda_1 t} \sum_{i \in \{1,\cdots,D\}} C_{1,i}^\partial \Big) = n \lambda_1 e^{\lambda_1 t} \sum_{i \in \{1,\cdots,D\}} \hat{C}_{1,i} = n \lambda_1 e^{\lambda_1 t} \hat{C}_1, \tag{C23}$$

as desired.

□

## C.4 Optimal power of the test for saturation

We know that the likelihood-ratio test is the most powerful $\alpha$-level test, as proved by Neyman and Pearson (1933). Given the log-likelihood $f(t)$ of branch $AB$ having length $t$, if the MLE is $\hat{t} < \infty$, the likelihood-ratio test compares the null hypothesis $t^* \to \infty$ versus the alternative hypothesis $t^* = \hat{t}$ using statistic $f(\hat{t}) - f_\infty$, or more explicitly

$$\text{"Reject } t^* \to \infty \text{ if } f(\hat{t}) - f_\infty > c\text{"}, \tag{C24}$$

where $c \in \mathbb{R}_{>0}$ is chosen so that $\mathbb{P}(f(\hat{t}) - f_\infty > c \mid t^* \to \infty) = \alpha$.

Assuming that $\hat{t}$ is large enough, we can use the approximation $f(\hat{t}) \approx f_\infty + \hat{C}_1 n e^{\lambda_1 \hat{t}}$ of Prop. C.5. This gives the test

$$\text{"Reject } t^* \to \infty \text{ if } \hat{C}_1 > e^{-\lambda_1 \hat{t}} c/n\text{"}, \tag{C25}$$

where $c \in \mathbb{R}_{>0}$ is chosen so that $\mathbb{P}(\hat{C}_1 > e^{-\lambda_1 \hat{t}} c/n \mid t^* \to \infty) = \alpha$. Setting $c' := e^{-\lambda_1 \hat{t}} c/n$, it is clear that the one-sided test for saturation using the coherence coefficient $\hat{C}_1$ achieves optimal power for long branches.

# Bibliography Supplementary Information

[Supp20] Hug, L., Baker, B., Anantharaman, K., Brown, C., Probst, A., Castelle, C., Butterfield, C., Hernsdorf, A., Amano, Y., Ise, K., Suzuki, Y., Dudek, N., Relman, D., Finstad, K., Amundson, R., Thomas, B., and Banfield, J. (2016). A new view of the tree of life. *Nature Microbiology*, 1:16048.

[Supp2] Neyman, J. and Pearson, E. (1933). On the problem of the most efficient tests of statistical hypotheses. *Philosophical Transactions of the Royal Society of London Series A*, IX(231):289–337.

[Supp38] Reden, F. (2023). EvoNAPS: A database for natural parameter settings of evolutionary models. Master's thesis, University of Vienna. https://doi.org/10.25365/thesis.76008.
