# Supplementary material for: When the Past Fades: Detecting Phylogenetic Signal with SatuTe
Source: Mol Biol Evol. 2025 May 27;42(5):msaf090. doi: 10.1093/molbev/msaf090 (PMC12108095; doi:10.1093/molbev/msaf090)
Supplement: msaf090_Supplementary_Data [file msaf090_supplementary_data.zip › SuppFigure1_effect_short_branches.pdf]

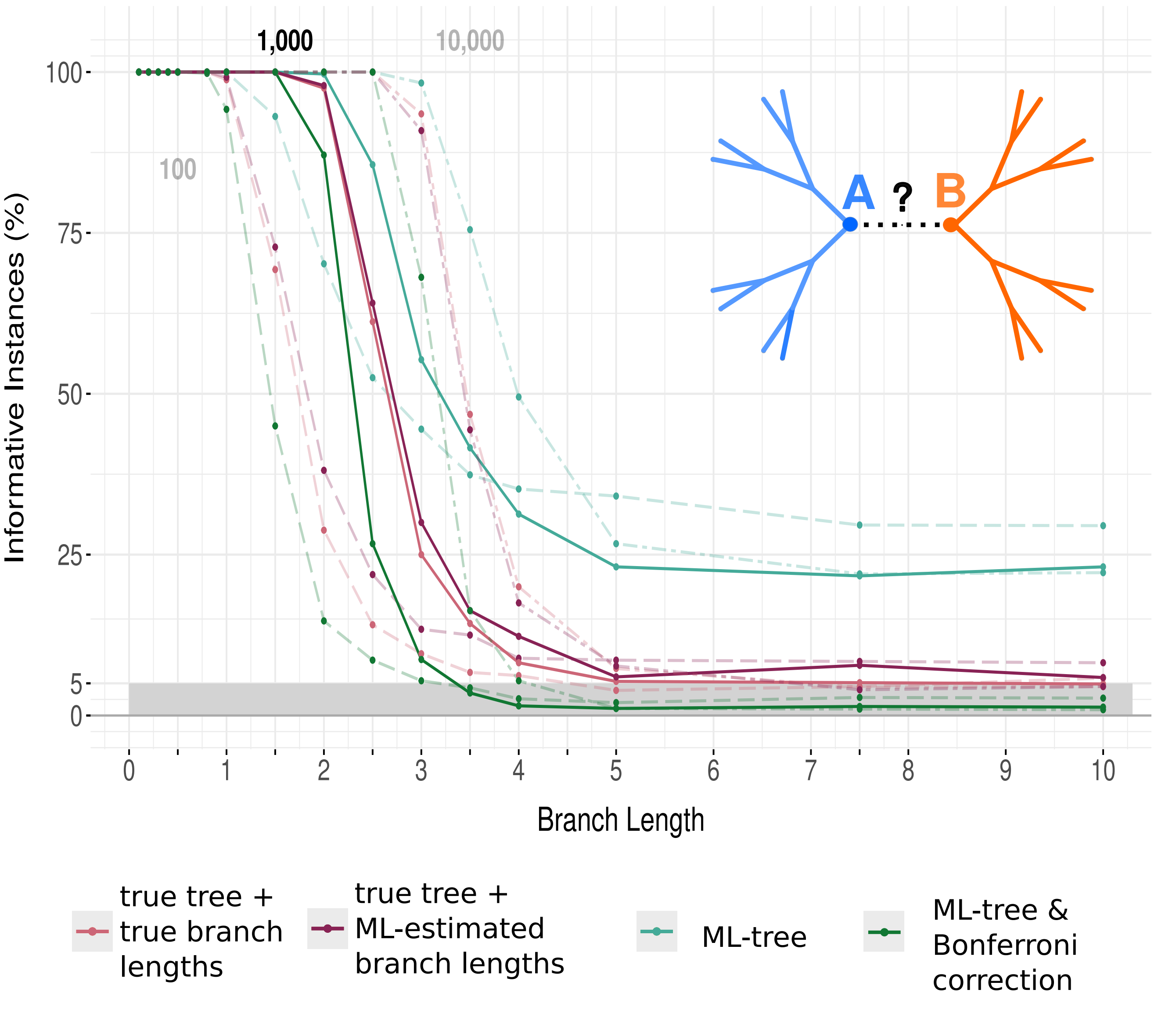

1,000
10,000
100
100
75
50
25
5
0
Informative Instances (%)
A
?
B
0
1
2
3
4
5
6
7
8
9
10
Branch Length
true tree + true branch lengths
true tree + ML-estimated branch lengths
ML-tree
ML-tree & Bonferroni correction
