# Supplementary figures and images for: When the Past Fades: Detecting Phylogenetic Signal with SatuTe
Source: Mol Biol Evol. 2025 May 27;42(5):msaf090. doi: 10.1093/molbev/msaf090 (PMC12108095; doi:10.1093/molbev/msaf090)

Density

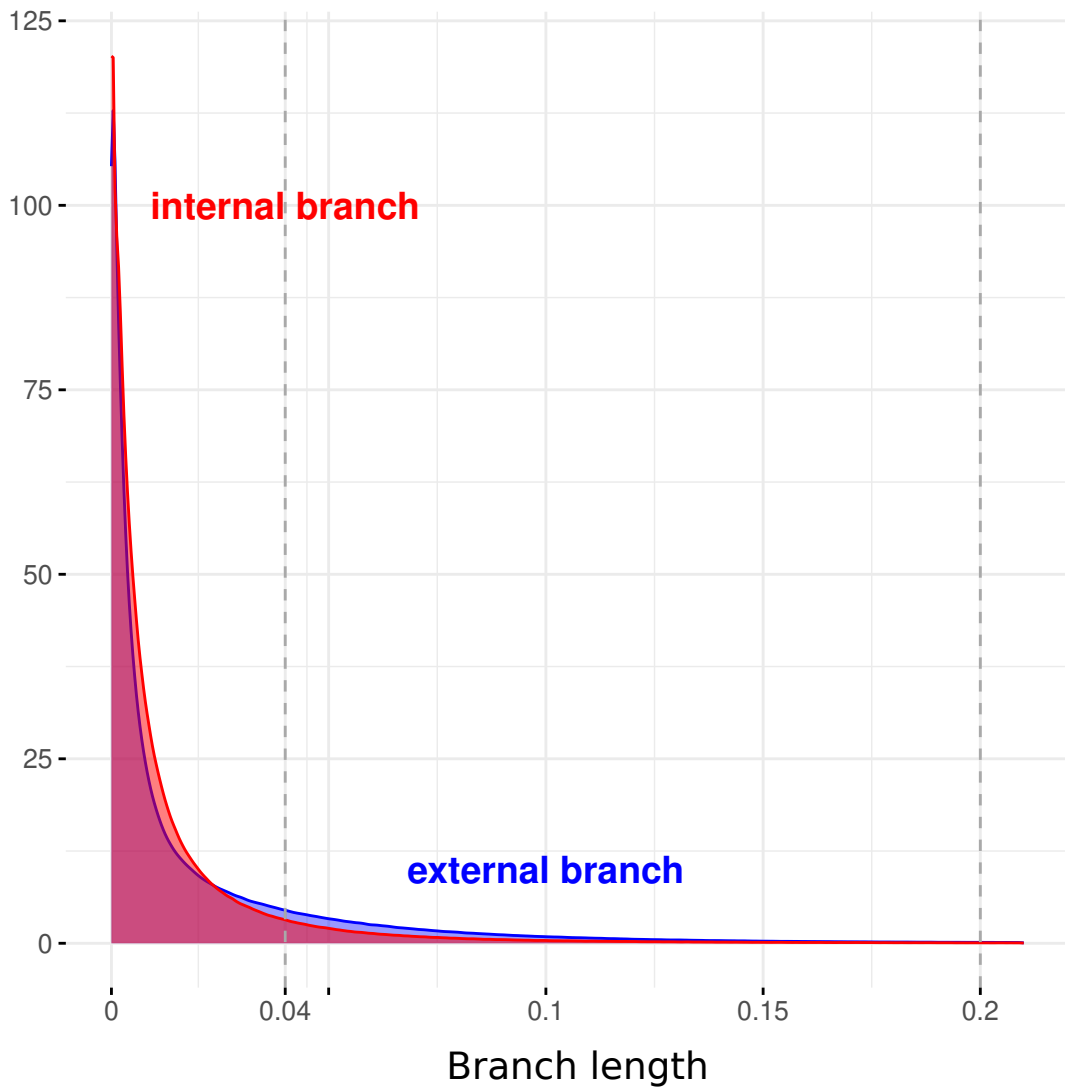

**internal branch**

**external branch**

Supplement: msaf090_Supplementary_Data [file msaf090_supplementary_data.zip › SuppFigure2_EvoNAPS_branch_length_distribution.pdf]

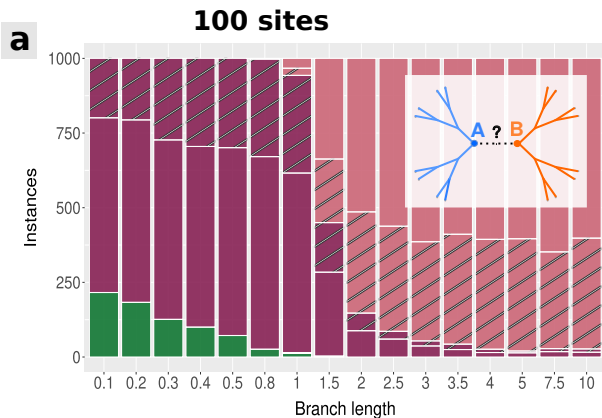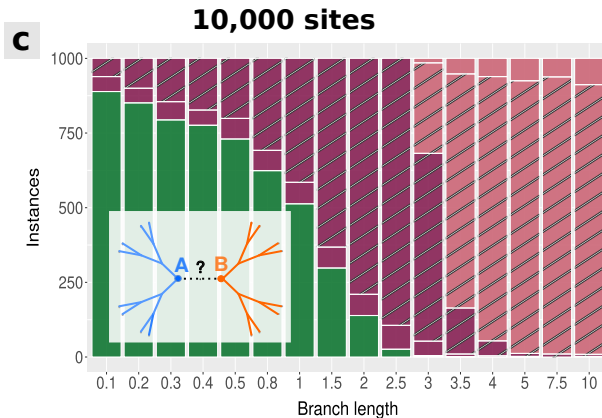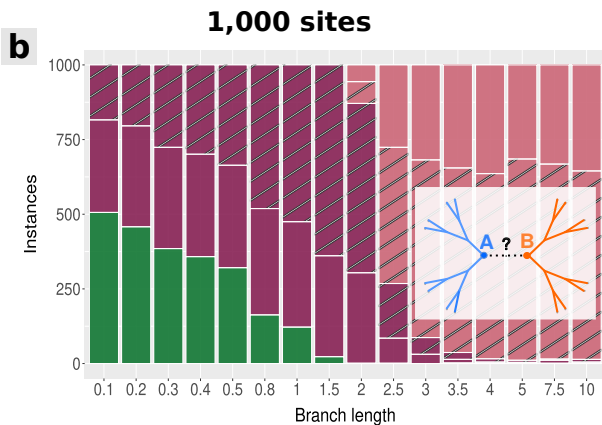

Supplement: msaf090_Supplementary_Data [file msaf090_supplementary_data.zip › SuppFigure3_saturation_accuracy.pdf]

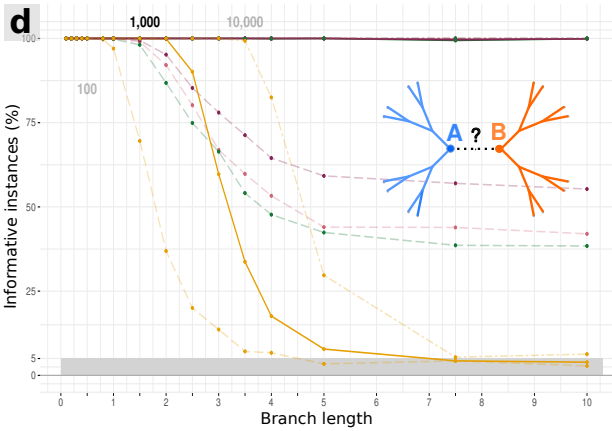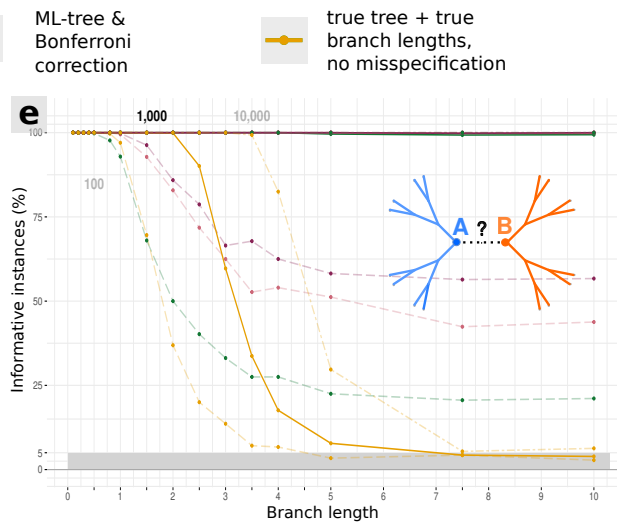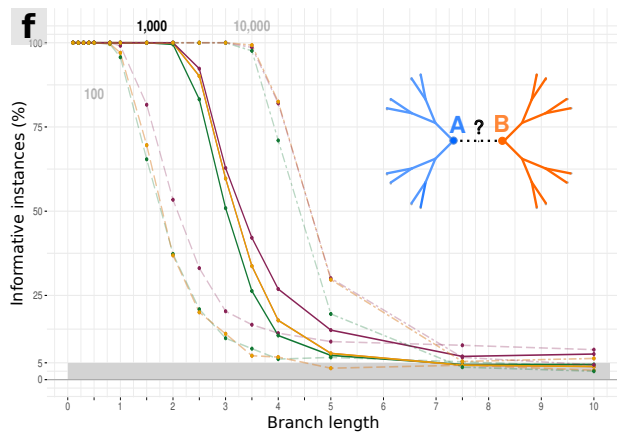

Supplement: msaf090_Supplementary_Data [file msaf090_supplementary_data.zip › SuppFigure4_simulated_data_misspecification.pdf]

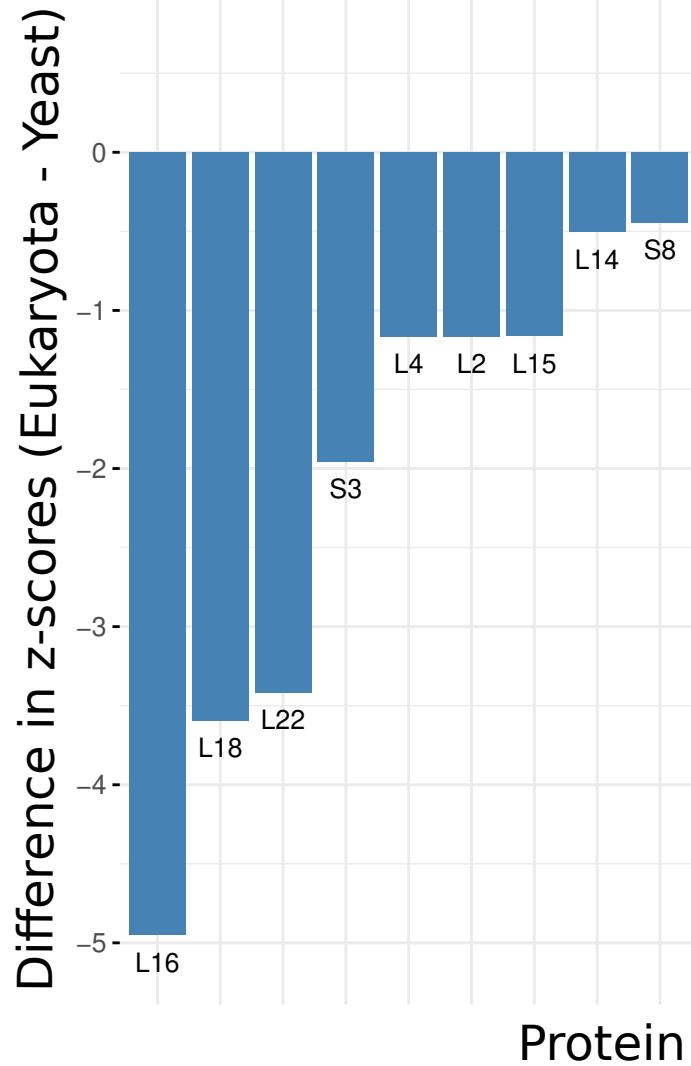

Supplement: msaf090_Supplementary_Data [file msaf090_supplementary_data.zip › SuppFigure5_protein_based_comparison_Yeast_Eukaryota_diff_zscores.pdf]
